# Perceptual lengthening of visual time throughout learning of hand-reaching actions

**DOI:** 10.1016/j.isci.2026.117993

**Published:** 2026-10-25

**Authors:** Nicola Binetti, Federico Mancinelli, Marco Zanon, Domenica Bueti

**Affiliations:** 1 International School for Advanced Studies (SISSA), Trieste, Italy; 2 Department of Systems Medicine, Università degli Studi di Roma Tor Vergata, Rome, Italy; 3 TRA Life and Health, University of Bonn, Bonn, Germany

**Keywords:** time perception, motor learning, motor planning, hand reaching, goal-directed action, electroencephalography, EEG, representational similarity analysis, RSA, sensorimotor integration, decision-making

## Abstract

Timing is intimately woven into human cognition and behavior, yet the dynamic interplay between timing and action remains poorly understood, particularly during goal-directed action learning. Using combined behavioral, electroencephalographic, and modeling approaches, we investigated how perceptual time changes as participants gained experience in a goal-directed reaching task requiring different degrees of action planning. Participants judged brief visual durations while preparing hand movements toward visual targets. Subjective time expanded during action preparation, which gradually increased across trials mirroring gradual improvements in action readiness. Representational similarity analysis revealed dissociable neural responses linking action and timing: action planning influenced time encoding during timed stimulus presentation, whereas broader motor engagement shaped subsequent time decoding following stimulus presentation. These findings extend previous accounts of action-dependent changes in perceived time, showing that the interaction is dynamic, evolving with changes in action readiness, and is mediated by dissociable neural responses during time encoding and decoding stages.

## Highlights

• Perceived visual time expands with reaching preparation and action plan specificity
• Perceptual time expands alongside improvements in action readiness across trials
• Distinct neural activity patterns link action planning to duration encoding and decoding

## Graphical abstract

**Figure:**
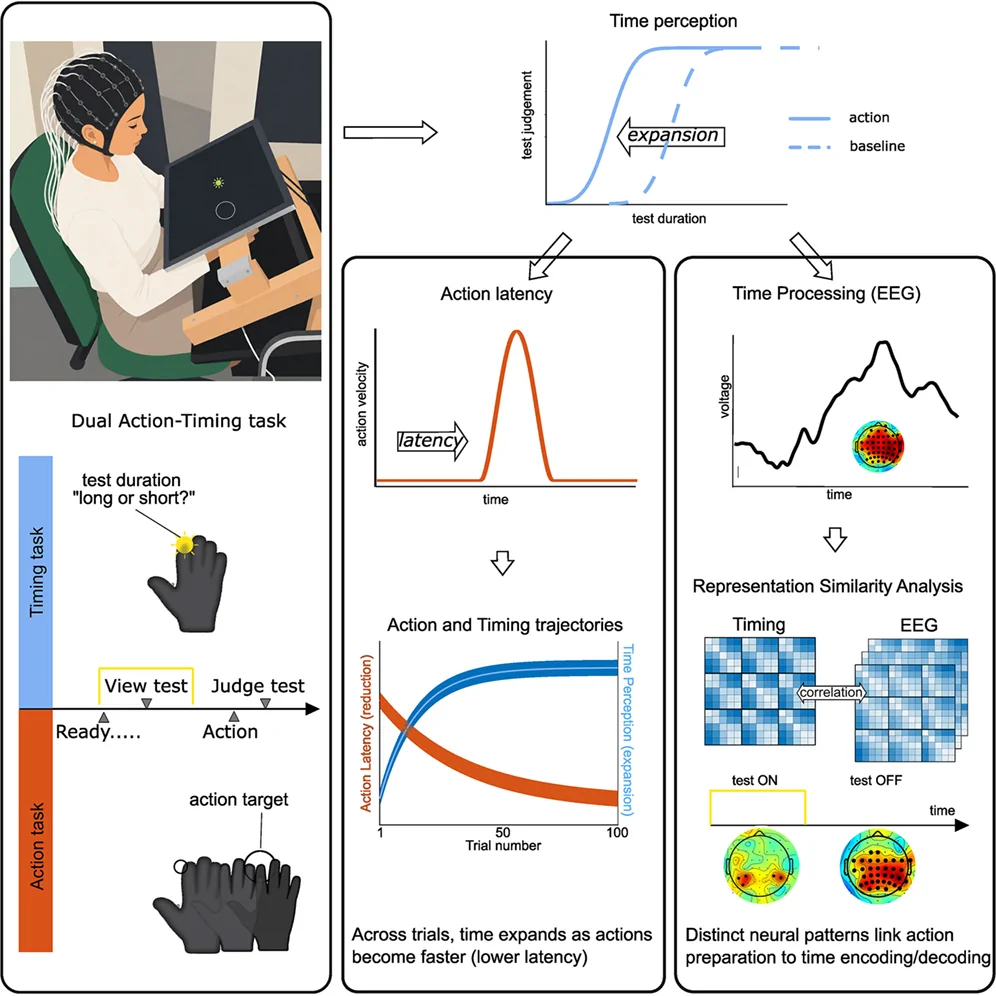

## Teaser

Behavioral neuroscience; Sensory neuroscience; Cognitive neuroscience; Psychology

## Introduction

Timing is pivotal to cognition and behavior, allowing people to predict events based on temporal expectations, to infer causality based on proximity of events in time, and to successfully interact with the environment. Humans exhibit highly precise time estimation, especially in millisecond to second ranges,^1^^,^^2^ despite the absence of a single sensory or neural system uniquely dedicated to timing. Converging evidence suggests that the brain bypasses these constraints by exploiting motor control circuitry, endowed with millisecond-level temporal precision required for action control.^3^ Indeed, at its core, action control can be viewed as a temporal problem, given that actions ranging from walking to striking a match-winning penalty kick hinge upon precisely scheduled muscle activations.

A motoric basis of time is supported by neural and behavioral evidence. Brain studies highlight a spatially distributed timing architecture, rooted in a motoric “core timing network,”^4^^,^^5^ comprising the supplementary motor area (SMA),^6^ the basal ganglia (BG), and cerebellum.^7^^,^^8^ This core set is augmented by auxiliary cortical and subcortical areas that are flexibly recruited based on task demands.^9^^,^^10^ Timing computations are similarly distributed in the frequency domain, carried out by oscillatory neural activity involved in a breadth of motor-related computations, such as action scheduling^11^ (4–7 Hz theta band), sensorimotor integration^12^ (theta and 8–12 Hz alpha/mu), and prediction^13^^,^^14^^,^^15^ (13–30 Hz beta). A large body of behavioral studies also reveals a significant overlap between timing and action, where perceptual time is significantly distorted in proximity of behavior. Action-timing interactions can be parsed based on when timing is sampled relative to action. Time is expanded as we prepare actions,^12^^,^^16^^,^^17^ whereas it is compressed while we carry out responses,^18^^,^^19^^,^^20^ and again expanded in the ensuing period where we evaluate behavioral consequences.^21^^,^^22^ These effects interact with the way actions are carried out, modulated by direction,^23^ force,^24^^,^^25^ frequency,^26^ and speed,^27^ generally reflecting a compatibility of action and perceptual magnitudes, with stronger, faster, and larger movements expanding perceived time.^28^^,^^29^ Studies also reveal that actions affect perceptual timing precision, with timing estimates sharpened during the execution of voluntary^30^ and involuntary^31^ behaviors.

While highlighting a complex and multifaceted relationship, timing-action interactions have mostly been explored through paradigms that overlook critical aspects of motor function, namely, that actions are frequently goal driven, and action control is gradually refined through practice. Actions involve goals (such as grabbing a set of keys laying on a table), plans that jointly specify motor instructions and predicted sensory consequences of behavior (planning a reach and what we expect to feel when touching the keys),^32^ and error signals (accuracy), critical for monitoring and course-correcting behavior.^33^ Error signals are additionally instrumental to action learning, improving performance through gradual refinement of motor plans and control strategies.^34^^,^^35^ However, most action-timing studies lack precisely defined action goals with associated errors, with participants performing behaviors with little or minimal constraint on movement parameters, such as moving in a given direction,^23^^,^^36^ moving fast,^27^ freely exploring an action space,^37^ and involving simple actions operating at ceiling-level performance such as touching a screen (Hagura et al., 2012), or saccading to a visual target.^38^ Tasks lacking explicit goals, or providing limited headroom for improvement, do not allow tracking changes in action performance across time and measuring the impact of these changes on perceptual time. Also, most action-timing studies provide static snapshots of timing performance, in terms of average timing estimates observed in an experimental condition relative to controls. However, action control is refined over time, and given a motoric foundation of timing, one should expect variations in time perception throughout goal-driven motor tasks. Recent studies provide a significant stride forward showing that rhythmic motoric structure spontaneously emerges as individuals gradually refine skills at a throwing task,^39^ suggesting that implicit timing is an emergent property of goal-driven motor learning and that implicit timing rehearsed in a motor task generalizes to perceptual contexts.^40^

Through combined behavioral, brain recording (electroencephalography—EEG) and modeling approaches, we aimed to study how conscious (explicit) timing estimates are progressively shaped throughout goal-directed action control. Participants judged the duration of brief visual stimuli presented during an interval preceding rapid hand reaches toward visual targets. Action preparation was manipulated by varying whether target locations were specified before movement onset, allowing participants to prepare a specific action plan, or only revealed at the Go signal, requiring action selection immediately before movement initiation.

Building on previous work showing that perceptual time expands during action preparation, particularly when actions are planned toward known target locations,^16^^,^^17^ we investigate behavioral and neural indices of action planning specificity on perceptual expansion of time. First, we compared conditions in which participants could prepare hand reaches toward visually cued target locations before movement onset with conditions in which the target location was not cued prior to movement onset, preventing the preparation of a specific action plan. We hypothesized greater time expansion for visually cued actions, building on evidence that perceptual time expands with the degree of action planning,^16^ and expected this modulation to be reflected in EEG correlates of subjective duration observed during time decoding.^41^ Second, we examined whether goal-related spatial features (reach direction and distance) specified in the motor plan influence perceptual and neural timing computations. Based on previous reports that action direction and movement distance distort perceived time,^22^^,^^23^ we hypothesized relative compression of time for rightward reaches and greater time expansion for reaches covering larger distances. Finally, given that timing processes are dynamically linked to action,^3^^,^^39^^,^^40^ we asked whether time expansion during planning evolves as motor performance is refined throughout goal-driven learning. We therefore tracked perceptual timing across the course of learning, hypothesizing that time expansion would change systematically across trials as action planning and control were progressively refined, leading to either gradual increase of expansion or reduction in expansion (time is recalibrated by action).

## Results

An acoustic Go signal prompted participants to perform a ballistic reach aimed at circular targets displayed on a monitor placed horizontally above their dominant hand. On-screen target locations varied based on direction (Directional task: left or right) or distance (Distance task: rightward near or far), relative to fixation. Reaching behaviors required rapid horizontal shifts in index position and were performed and tracked on a Plexiglass surface placed under the monitor (Figures 1A and 1B). A visual depiction of the index landing point was displayed on the monitor at the end of each trial, providing accuracy feedback. Targets were either shown (Cued location among two alternative locations) or not shown (non-cued location among two alternatives) during the action foreperiod preceding the Go signal (Figure 1D). Thus, Cued trials allowed for preparing a specific reach prior to the Go signal, whereas non-cued trials, where target presentation coincided with the Go signal, additionally required participants to select among plans required in the current task (left or right in Directional task; near or far in Distance task; Figure 1C). During the action foreperiod, participants additionally judged the duration of sub-second visual Test stimuli (0.64–1.24 s) presented at central fixation. Judgments were made relative to a reference Standard duration (0.84 s), which was presented at the beginning of the testing session, and refreshed throughout the experiment (method of single stimuli). The timing task was also performed without motor responses in matched intervals, requiring delayed timing judgments following the Go signal (sensory control). This design allowed us to address three questions. First, comparing Cued and Non-cued trials tested whether perceptual time expansion depends on the specificity of action planning. Second, manipulating target direction (left/right—Directional task) or distance (near/far—distance task) tested whether goal-related spatial features of the planned action influence timing estimates. Finally, by tracking behavior and timing throughout trials, we examined whether action-timing interactions evolve as motor performance is progressively refined. To dissociate effects of planned motor commands from those of their predicted visual consequences, we additionally manipulated the mapping between hand movements and cursor motion (Congruent vs. Incongruent). In the Directional task, leftward hand movements produced either leftward (Congruent) or rightward (Incongruent) cursor motion, whereas in the Distance task, short hand movements produced either short (Congruent) or long (Incongruent) cursor displacements. This manipulation allowed us to distinguish the influence of planned action features from that of their predicted sensory consequences on perceptual timing.

**Figure 1:**
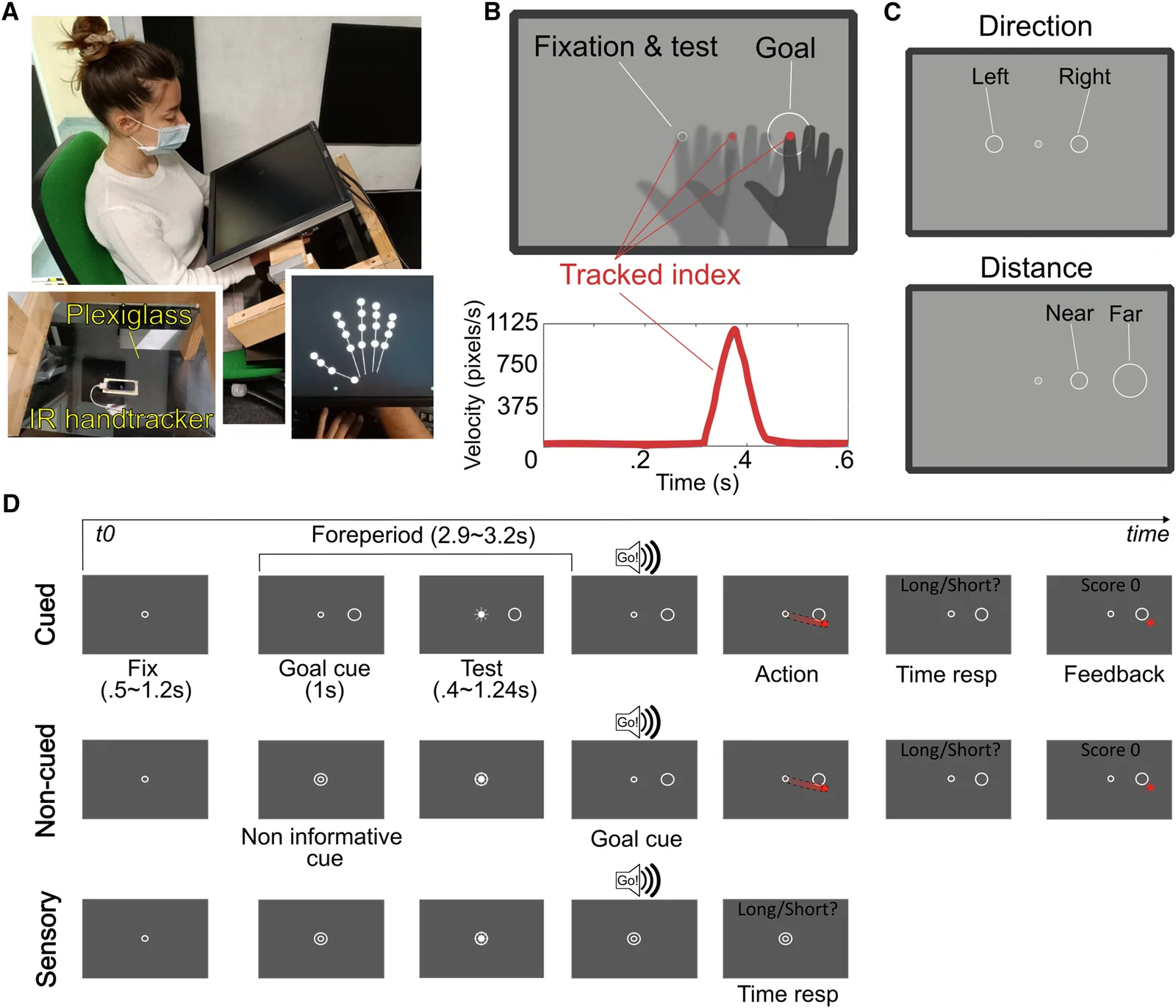
Task setup and experimental conditions (A) Participants performed ballistic horizontal hand movements on a Plexiglass surface, tracked by an infrared (IR) camera. (B) Movements were aimed at goal locations displayed on a monitor that occluded participants’ hands, in cued and non-cued task conditions. Participants’ indices remained stationary under the fixation point during matched passive observation conditions (sensory). (C) On-screen target locations varied based on direction (directional task: left or right) or distance (distance task: rightward near or far). (D) During a variable foreperiod (2.9–3.2 s), participants were shown a goal location for 1 s in cued trials (right or left in the direction task; right near or far in the distance task), after which the fixation point lit up for a variable amount of time, providing a test duration (0.4–1.24 s). The remaining interval until the end of the foreperiod was a variable delay, ensuring that the go signal never overlapped with the test stimulus. An acoustic beep (Go!), coinciding with the presentation of the goal in the non-cued condition, signaled the end of the foreperiod, requiring participants to sequentially perform the hand movement (cued and non-cued; index position occluded) and the timing judgment (all conditions). The final position of the index was revealed at the end of the trial, providing feedback on reach accuracy. A score (1–100) was provided for actions landing within the goal area, proportional to the distance from the goal center.

Overall, we found that visual time was perceptually expanded by action planning. On average, timing estimates expanded prior to action onset in Cued compared to Non-cued actions and Sensory conditions. However, trial-wise models of timing responses revealed that both action conditions (Cued and Non-cued) showed progressive expansion of perceptual time across trials as action latency (the time required to initiate a reach following the Go signal, reflecting readiness to enact a motor plan). Representational similarity analysis (RSA) of EEG data showed that these distortions were associated with neural activity during both the encoding of duration information, as the Test stimulus unfolded, and the subsequent decoding period following stimulus offset, when temporal information is read out to inform the duration judgment. Encoding-related effects were associated with action planning, whereas decoding-related effects were associated with more general action preparation processes. We detail these findings in the following sections.

### Average timing performance—Action planning biases perceptual time

We compared measures of timing bias (point of subjective equality—PSE: Test duration that is perceptually matched to the Standard) and sensitivity (just noticeable difference—JND: detectable difference between Test durations) across Conditions (4 Cued conditions + Non-cued & Control), within both Directional and Distance tasks (Figure 2A). Linear mixed effects analysis on PSEs showed a perceptual expansion of time across Cued conditions relative to both Non-cued trials and sensory Controls, in both Directional (F(5,95) = 6.86, *p* = 1.65e^−05^; R^2^ = 0.06, conditional R^2^ = 0.79; Figure S1) and Distance tasks (F(5,95) = 3.15, *p* = 0.01; R^2^ = 0.04, conditional R^2^ = 0.7; Figure S2). Therefore, when averaging across trials, Cued conditions, where motor plans were ready to be executed at the Go signal, were associated with a perceptual expansion of time, relative to Non-cued reaches that required plan selection at the Go signal, or matched controls that did not engage the motor system. Action features (left/right or near/far reaches) and hand-to-screen space mappings (Congruent or Incongruent mappings: see STAR Methods) had no modulatory effect on timing estimates (see Figures S14 and S15).

**Figure 2:**
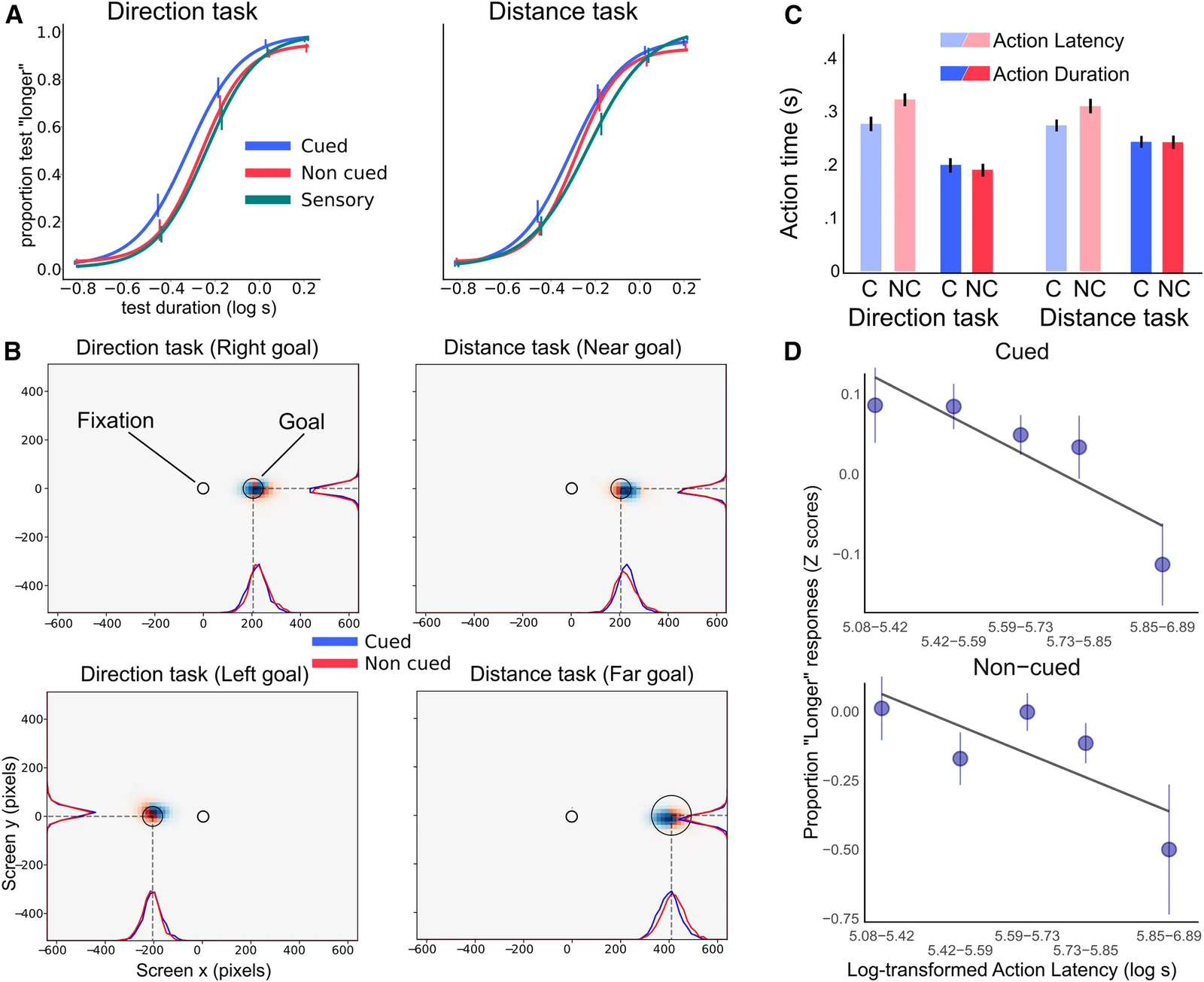
Timing and reaching action task performances (A) Psychometric fits of test “longer” responses as a function of test duration, across experimental conditions. Time is perceptually expanded in cued (C) relative to non-cued (NC) and sensory conditions, across both direction and distance tasks. (B) Density of index finger landing points in screen space across experimental conditions and tasks (also projected onto individual axes). Action precision, quantified as the distance between landing points and goal center (which also informs reinforcement schedule), did not differ between C and NC actions and between tasks, when accounting for linearly scaled errors for far goals in the distance task. (C) Reaching action latency (time required to initiate action, from go signal to movement onset) and duration (time required to complete action, from action onset to offset). Action latency was longer in NC than in C actions, consistent with the greater goal location uncertainty in the NC condition (see Figures S3 and S4). (D) Perceived time (proportion “longer”) as a function of action latency. Shorter latencies were associated with longer duration percepts. Error bars represent standard error of the mean (SEM).

JNDs showed no significant difference in sensitivity to duration across conditions, in both Directional (F(5,95) = 0.43, *p* = 0.82; R^2^ = 0.01, conditional R^2^ = 0.24) and Distance tasks (F(5,95) = 2.18, *p* = 0.06; R^2^ = 0.06, conditional R^2^ = 0.3). Thus, timing task difficulty did not significantly differ across tasks (Directional or Distance) and conditions.

### Changes in time perception driven by action readiness

We modeled timing judgments on a trial-by-trial basis in Cued and Non-cued conditions to capture the dynamic relationship between changes in action performance and changes in time perception.

(A separate analysis is reported in the supplemental information in which this same analysis is carried out in the two conditions separately; supplementary analysis 1.) We extracted action features from right index position time series, such as action latency, mean velocity, reaching error (the difference between the index’s final position and the goal center, reflecting reach precision), and distance traced (product of action duration and mean velocity). Individual factors for each Test duration were inserted into the model so that we could assess the contribution of action features in explaining the residual variance.

Analysis revealed a strong influence of log-transformed action latencies on timing judgments (b = −0.79, 95% CI [−0.97, −0.62]). Shorter latencies were associated with an increase in “Test longer” responses, i.e., trials in which participants judged the Test stimulus as longer than the reference duration, indicating greater perceived duration (time expansion) (Figures 2C and 2D; reaching performance did not vary across conditions Figure 2B). Additionally, we observed significant effects of both tasks (Direction and Distance) and conditions (Cued and Non-cued) on duration judgments. The Distance task resulted in greater time contraction (b = −0.20, 95% CI [−0.29, −0.11], relative to Direction), and Non-Cued condition resulted in greater time contraction (b = −0.30, 95% CI [−0.41, −0.19], relative to Cued). Independent of task and cue conditions, log-transformed action latencies consistently decreased across trials (b = −0.05, 95% CI [−0.06, −0.03]), while “Test longer” responses increased correspondingly (b = 0.29, 95% CI [0.12, 0.44]). Given these opposite changes, we next explored whether the rate of decrease in action latencies could predict the increase in perceived time expansion at the participant level. To address this, we constructed simple linear models of the trial-by-trial trajectories for both perceived duration and action latencies, incorporating participant-level random effects. To enhance statistical power for this individual-level analysis, data from the two tasks and cue conditions were combined, providing a single, robust random-effect estimate per subject.

Participants’ individual parameters for rate of change (alpha parameters) in timing judgments across trials negatively correlated with rates of change in action response log latencies (r = −0.32, *p* = 0.02, 95% CI [−1, −0.057]; Figure 3A). Thus, positive and negative values indicate opposite directions of change over trials, whereas the absolute value indicates the magnitude of change; +1 and −1 are equal in size but opposite in direction. Our interpretation here is directional: participants with larger repetition time (RT) reductions over trials (more negative RT slopes) showed more negative changes in bias.

**Figure 3:**
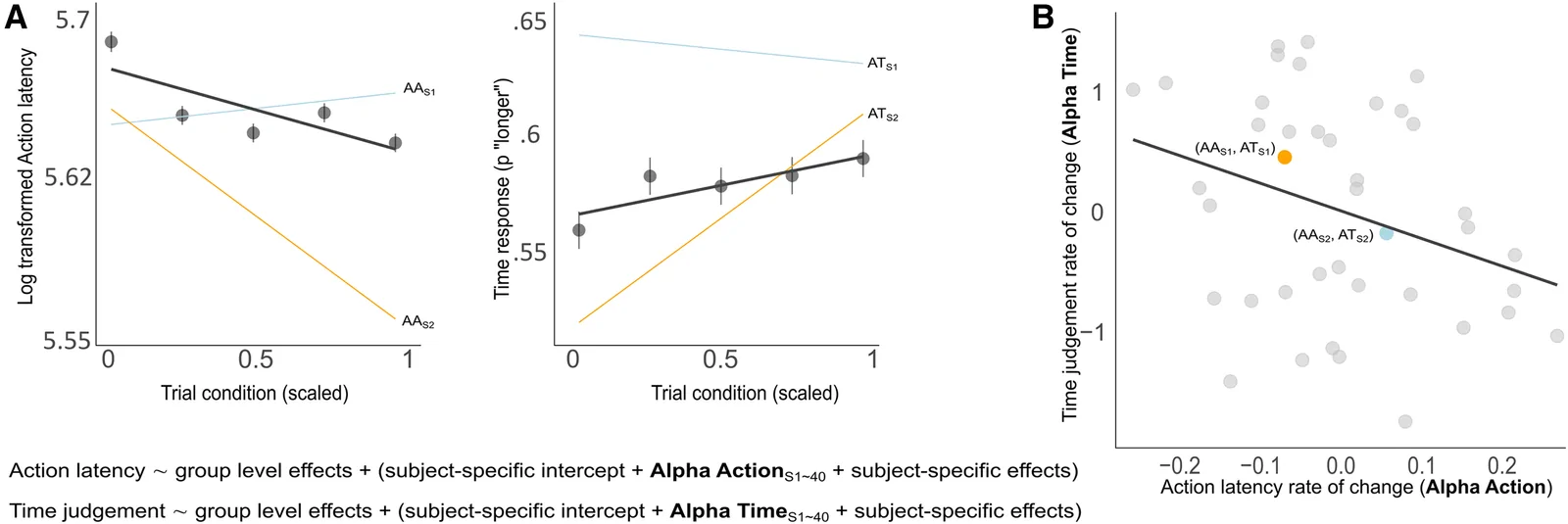
Modeling changes in timing judgments and action latency across trials (A) Changes in Action latency (left plot) and Time judgments (“longer” judgments; right plot) as a function of trial number throughout cued and non-cued trials (scaled to account for differences in trial numbers). Thick dark lines represent the group average, while colored lines represent 2 participants, showing opposite trends relative to the group average. Action latency and time judgments were separately modeled as a function of test duration, trial number within each condition (t_c_, capturing global changes occurring across all cued/non-cued condition trials—significant), and trial number within blocks of 20 trials (t_c,b_, capturing local changes associated with switches between task conditions—non-significant). Participants were modeled as random slopes, providing individual parameters for rate of change in action latency (αAction,I = participant i’s random slope for t_c_ in the log-latency model) and time judgments (αTime,i = participant i’s random slope for t_c_ in the time-judgment model) across trials. See precise formulation in STAR Methods. (B) αAction and αTime parameters across participants, representing subject-specific slope estimates extracted from the mixed-effects models (i.e., participant’s estimated effect of scaled trial number within each condition on [*x* axis] log action start latency and [*y* axis] bias [“longer” responses]). Participants show inversely related rates of change in action latency and time judgments (greater decreases in action latency relate to greater increases in time perception, over trials). Error bars represent SEM.

Participants with greater rates of decrease in action latency exhibited greater increases in “Test longer” responses across trials (Figure 3B). As a sanity check, to reduce the impact of changes in action latency occurring early in each condition, we additionally excluded data from the first block in both conditions, focusing only on later trials. We then re-fit the model, generating new rate of change parameter estimates. This preprocessing step yielded consistent results, suggesting that the observed relationship reflects a task-wide process rather than transient performance fluctuations occurring in early trials only (r = −0.31, *p* = 0.03, 95% CI [−1, −0.04]).

### EEG time encoding and decoding correlates

Evoked Reponse Potentials (ERPs) for time encoding and decoding were analyzed to identify the effect of test duration (5 test durations) and condition (sensory, non-cued, and cued) in both groups separately. No significant effects were observed during Time Encoding, for Test Duration, nor for Condition (Figure 4A, left column). Since any possible information about Test duration is provided during the selected interval for ERPs (in fact, epochs ranged from the Test onset to the offset of the shortest probe), no effects for Test Duration were expected, whereas lack of differences among conditions suggests that action planning does not seem to set a condition-specific “state” for time encoding.

**Figure 4:**
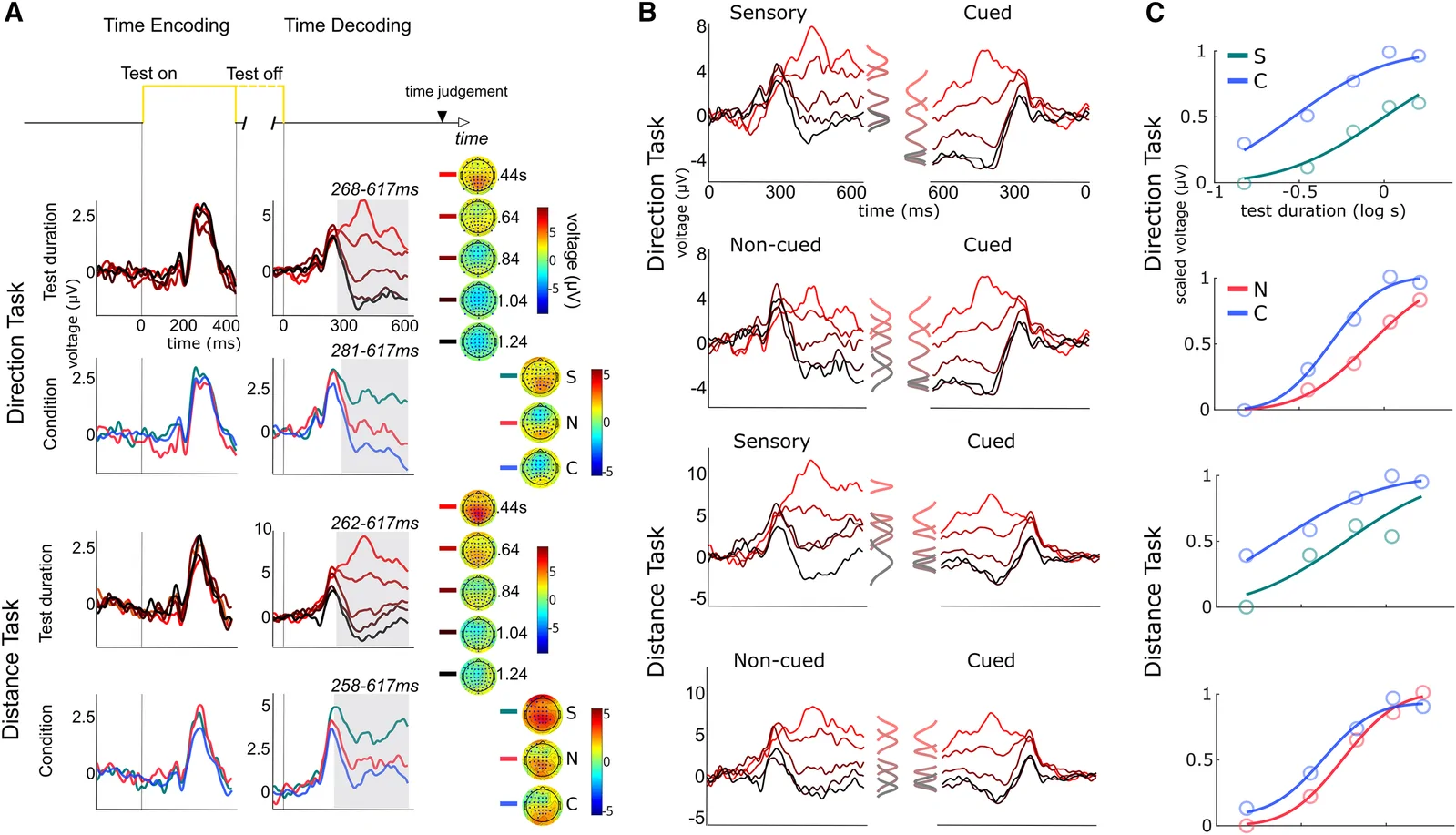
ERP analyses of visual duration encoding and decoding (A) Evoked neural responses time locked to Test duration onset and offset, indexing time encoding (online monitoring) and decoding (offline readout), respectively. The time encoding interval was constrained to the shortest tested duration (420 ms), while the decoding window corresponded to the shortest interval afforded by the variable foreperiod preceding the Go signal (617 ms). Gray shaded areas show intervals with significant differences between test durations (0.44–1.24 s, averaging across conditions) or conditions ([S]ensory/[N]on-cued/[C]ued, averaging across test durations), with corresponding topography of evoked signals on the right. (B) Side-by-side comparison of time decoding in cued vs. non-cued/sensory conditions (flipped on the time axis) at electrode Cz. Gaussian curves represent the average voltage ± scaled variability within time windows showing significant differences between tested durations (shaded regions in A), revealing a reduction of voltage across tested durations in cued relative to non-cued and sensory conditions. (C) Neurometric curves (cumulative Gaussian fit with lapse rates) of average normalized voltage within significant time decoding windows (shaded regions in A) as a function of tested duration, for (S)ensory, (N)on-cued, and (C)ued conditions.

Significant effects were observed during time Decoding in both groups. The effect is significant about 270 ms after Test offset, with a peak at about 350 ms (Figure 4A, right column), in central and parietal electrodes. ERP amplitudes depend on Test duration: the shortest duration (0.44 s) elicits a large positive potential, which diminishes with longer durations, showing an exponential decay (Figures S7 and S8). The result is in line with previous findings. Ofir and Landau (2022) showed that the EEG signal recorded in frontocentral electrodes about 300 ms after Test offset is a neural marker of the readout of the time perception system that during duration encoding accumulates evidence in favor of a “longer” response. To note, clusters of significant time points and channels were observed also for earlier latencies, but these are likely byproducts of the baseline correction. In fact, the 100-ms baseline interval for Decoding epochs was centered on Test offset, an interval that still includes ERP components elicited by the onset of the stimulus (see Figure S9; also present in Ofir and Landau [2022]).

The time Decoding effect of Condition is observed at frontocentral electrodes, where Non-cued and Cued conditions elicited more negative potentials than Sensory in both groups. These differences emerged around 300 ms following Test offset. Similar ERP amplitudes in Non-cued and Cued conditions, both distinct from Sensory, suggest that action readiness processes drive ERP components from this time point onward. This interval appears crucial for understanding the neural basis of the behavioral bias in time perception. However, it also marks the overlap of significant effects of both Probe Duration and Condition. At this stage, participants are preparing to move, especially in the Cued condition, where early cue presentation allows planning upcoming behavior. Consequently, ERP activity related to motor preparation may obscure subtle responses linked to time perception, which is behaviorally biased by motor preparation activity. To disentangle these overlapping processes, RSA was employed. Still, ERP comparisons across the three Conditions support the idea that Motor conditions (Cued and Non-cued) are well matched.

### Neural source of distorted percepts of duration

We aimed at identifying the neural source of perceptual distortions of time using RSA, identifying brain correlates based on similarly patterned behavioral and EEG activity (Figure 5).

**Figure 5:**
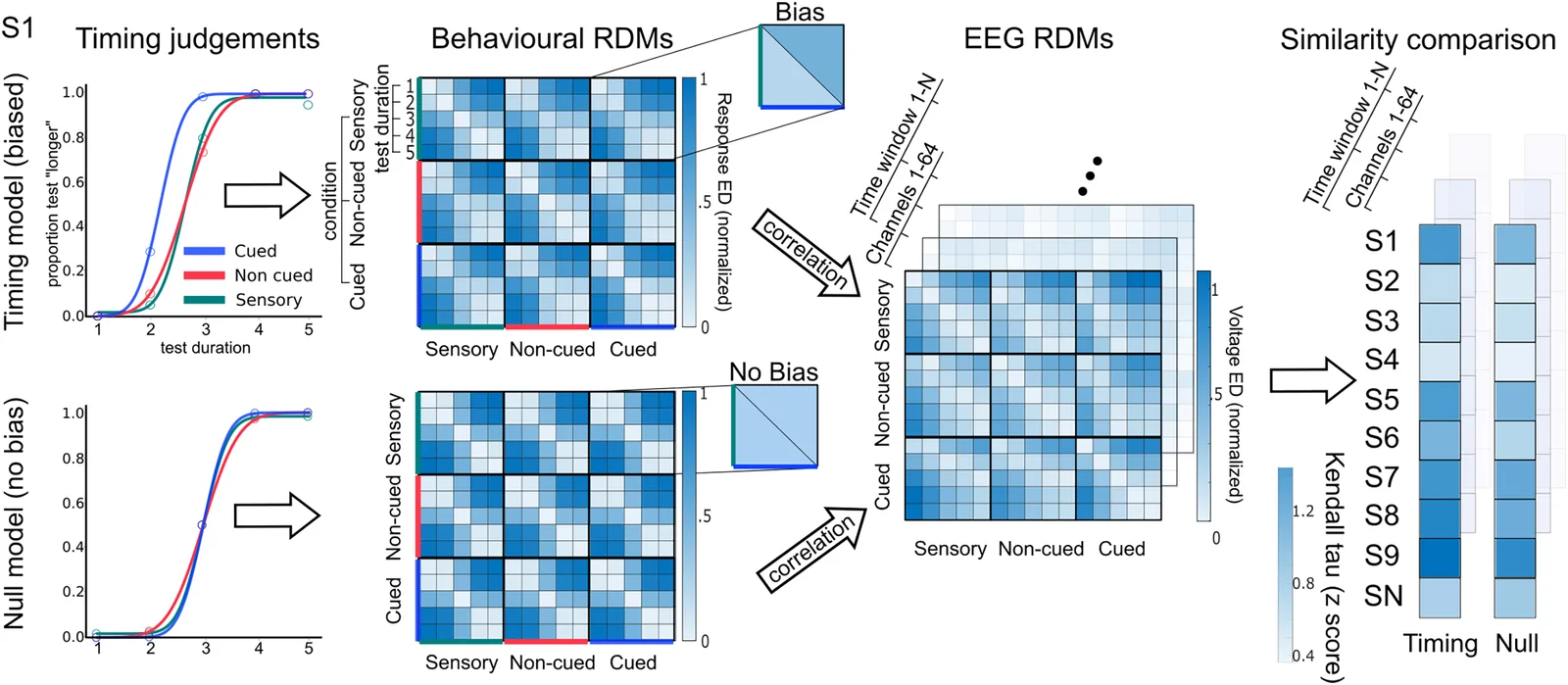
Representation similarity analysis pipeline used to reveal the neural source of the time perception bias observed in cued relative to non-cued and Sensory conditions Behavioral Representational Dissimilarity Matrices (RDMs) were built based on individual participant data (Euclidean distance in participant time judgments) or synthetic data (Euclidean distance in unbiased participant duration judgments—providing a null model), capturing the overall behavioral performance across tested durations and experimental conditions. Participant behavioral RDMs were compared to EEG RDMs (Euclidean distance in voltage, per channel and 50 ms time window) via Kendall Tau correlations, indexing pattern similarity (*Z* scores) between real/synthetic behavior and neural response. Real and synthetic pattern similarity *Z* scores were compared via *t* tests, revealing channel/s and time window/s at which behavioral/neural similarity was greater for biased relative to unbiased time perception, i.e., identifying where and when neural response more closely resembles biased time perception, relative to unbiased perception.

On average, perceived time was significantly lengthened in Cued relative to Non-cued actions and Sensory controls, for both Direction and Distance tasks. RSA results reveal that the perceptual expansion of time was associated with biased neural activity in time encoding and decoding, where duration information is respectively monitored and readout for perceptual judgments. Time encoding and decoding responded differently across experimental conditions (Figures 6A and 6B; see also Figures S10–S13). In the Direction task, time encoding was specifically impacted by the presence of a motor plan, as captured by contrasting Cued and Non-cued actions, reflected in responses at central (287–336 ms) and bilateral posterior electrodes (383–432 ms), following Test onset. In the Distance task, we also observed effects on time encoding: biased perception in Cued with respect to Non-cued actions was reflected in responses at central (50–100 ms) and temporo-parietal electrodes (336–383 ms), following Test onset. In the Direction task, time decoding was more generically linked to motor system engagement, captured both by Cued vs. Non-cued and Cued vs. Sensory contrasts, and reflected in responses at frontocentral (359–412 ms) and central electrodes (308–463 ms) respectively, following Test offset. The Distance task also showed similar patterns of activity in time decoding, which however did not reach significance.

**Figure 6:**
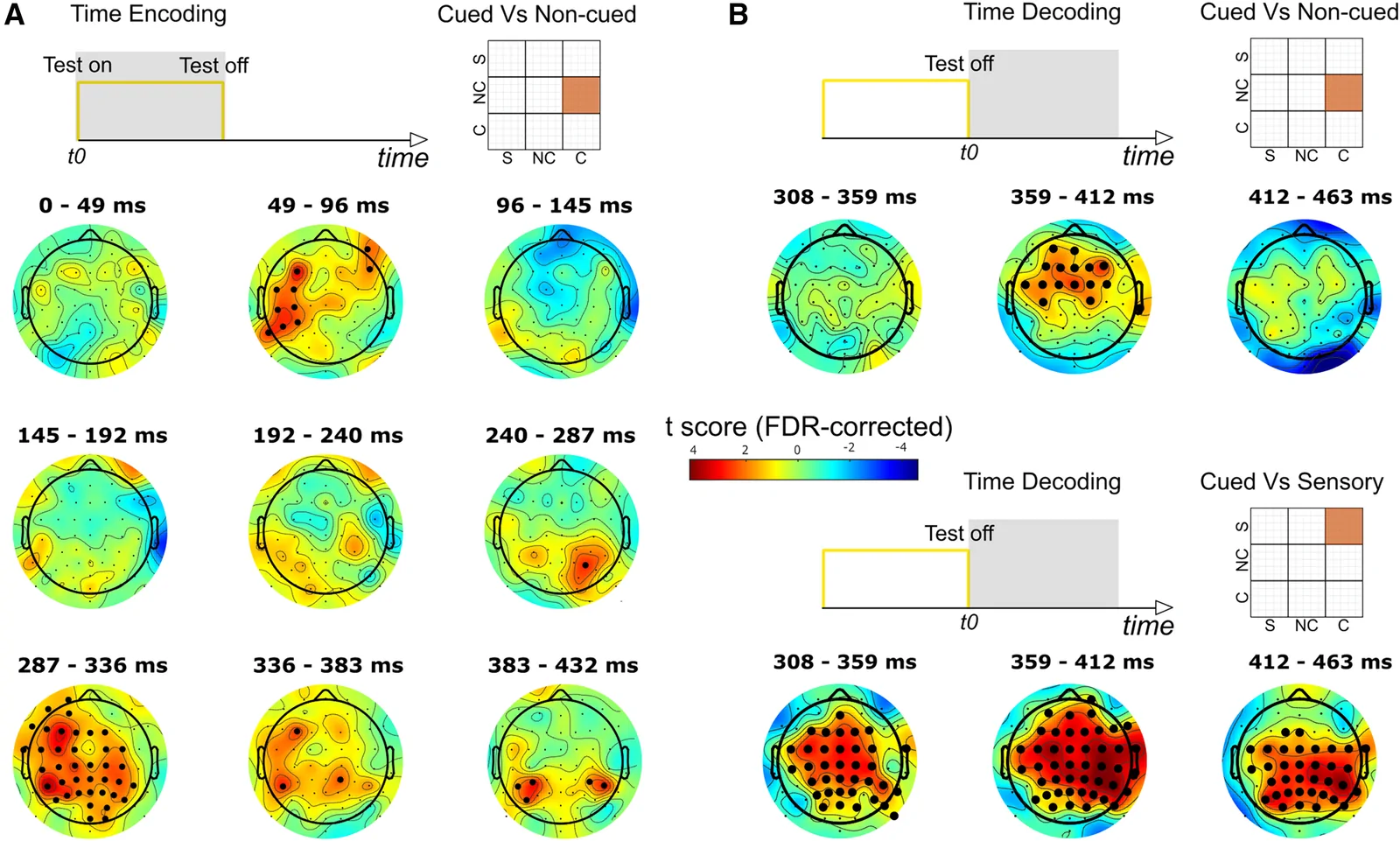
Representation similarity analysis contrasts RSA contrasts targeting RDM sub-quadrants, identifying neural correlates of perceptual distortions of time within time encoding and decoding windows. Perceptual biases in timing were reflected in neural activity throughout time encoding and decoding. (A) RSA revealed effects on time encoding specifically linked to action planning (cued vs. non-cued; summarizing effects in both direction and distance tasks, individually modeled in the supplemental information), within windows time locked to test onset. (B) Effects on time decoding more generically linked to motor system engagement (cued vs. sensory and cued vs. non-cued, only significant in direction task), within windows time locked to test offset.

## Discussion

Action and timing systems are deeply integrated in the brain. From a timing perspective, our ability of estimating duration engages motor systems,^42^^,^^43^ even in tasks requiring no overt behavior,^5^^,^^29^^,^^44^ and time perception is profoundly shaped by processes that straddle the line between action preparation, online control, and *post hoc* evaluation of action consequences.^19^^,^^23^^,^^25^^,^^45^ Importantly, the direction of these distortions depends on when temporal information is sampled relative to action: previous work has shown time expansion during action preparation, compression during action execution, and renewed expansion during post-action evaluation. The present study specifically focuses on the preparatory stage, building on studies by Hagura et al.^16^ and Iwasaki et al.,^12^^,^^17^ which reported temporal expansion under comparable task conditions. Action control in itself is a temporal (i.e., scheduling) problem.^3^^,^^46^ Logically, this implies that time perception exploits systems that are pre-equipped for precise timing.^47^ Action and timing are also inherently linked due to their dynamic nature. Actions are gradually honed through practice, driven by external goals and reward signals, while timing involves the integration of continuously unfolding duration evidence. Our findings highlight a dynamic interplay between action and timing that evolves alongside the refinement of action selection and planning processes, as reflected in faster initiation of voluntary actions.

### Action planning expands visual time

Comparison of average time perception biases (PSEs) across conditions showed time expansion during Cued trials compared to Non-cued and Sensory controls, thus suggesting that priming specific motor plans distorts subjective time. Previous studies have linked time expansion to preparation of hand reaches,^16^ button presses,^12^ or horizontal arm movements.^23^ Hagura et al.^16^ first investigated time expansion during action preparation. The authors included a matched non-motor dual-task condition (letter discrimination/timing task; Experiment 2) and showed that it had no effect on timing. In Experiment 3, they manipulated the degree of motor preparation by varying whether the target location was specified before or at movement onset, demonstrating that time expansion scaled with action preparation and thereby allowing attentional or arousal-based explanations to be ruled out. Our Cued and Non-cued conditions closely replicate this manipulation and its associated behavioral effect. An MEG study by Iwasaki et al.^12^ used the same dual-task structure and demonstrated that time expansion during action preparation arises from sensorimotor integration neural mechanisms. The authors show that visual stimulation and motor preparation each induce characteristic forms of alpha suppression and that their interaction during action planning explains the observed temporal expansion. Importantly, time expansion only emerged for filled visual intervals (continuous timed stimulus) and not for empty intervals (durations defined only by brief onset/offset transients).^17^ Thus, the timing effect requires ongoing sensory stimulation that is modulated by motor preparation, further excluding non-specific attentional accounts, that would affect timing irrespective of how visual information is delivered. Our study builds directly upon this literature by exploring how time expansion during motor preparation relates to action performance, and more specifically how these changes evolve throughout the task.

The interaction between motor and sensory systems conceptually aligns with the embodied cognition framework,^48^^,^^49^^,^^50^ where action possibilities (plans, motor repertoire, and affordances) are integrated with perceptual mechanisms. The temporal distortions observed during action planning can be viewed as a byproduct of interaction mechanisms that integrate motor and sensory signals^51^ and/or enhance processing of upcoming sensory consequences of behavior.^16^^,^^52^ Tomassini et al. showed that adaptation to tactile motion causes a contraction of the apparent duration of tactile stimuli and that this effect is abolished when participants perform voluntary movements before judging durations, restoring more veridical estimates of event time.^53^ This finding suggests that actions, or predictions of their sensory consequences, recalibrate or “reset” perceptual systems, also affecting the processing of temporal features embedded in the sensory input. Similarly, Hagura et al. showed improved visual discrimination of rapidly changing information prior to action execution, suggesting that sensory systems are enhanced to accommodate action-induced visual changes.^16^ This modulation may in turn influence temporal estimation, which is known to depend, at least in part, on the number of sensory changes processed.^54^^,^^55^^,^^56^

Spatial features of action have also been reported to alter perceived time. Tomassini and colleagues found relative time contraction and expansion preceding leftward and rightward hand movements, respectively.^23^ In contrast, we observed no effect of action spatial features (left vs. right or near vs. far reaches) or action-to-visual mappings (Congruent vs. Incongruent) on perceived duration. Our findings therefore suggest that perceptual time relates to temporal aspects of action preparation (i.e., when actions were initiated) rather than to non-temporal action parameters (i.e., where actions are directed). One possible explanation for the discrepancy with Tomassini et al. is that the two studies probed different stages of action preparation. Tomassini and Morrone sampled timing immediately before movement onset, whereas we probed timing over a longer interval preceding the Go signal, emphasizing earlier action selection and planning. Consistent with this interpretation, Iwasaki and colleagues, whose paradigm and tested durations more closely resembled ours, likewise found no modulation of timing by movement direction.

### Perceptual time is dynamically linked to changes in action latency

While on average Cued actions biased timing estimates relative to Non-cued/controls, trial-based timing models revealed a more nuanced relationship linking changes in action latency and time perception. Within participants, visual time progressively expanded as action latency decreased (faster reaction to Go), across both Cued and Non-cued trials. Given predefined target locations, Cued responses showed a marginal reduction in action latency across trials. Reduction in latency was more pronounced in Non-cued trials, likely because these offered a larger margin for process optimization as participants learned to cope with target location uncertainty. A possible strategy for dealing with target uncertainty in Non-cued trials can consist in simultaneously planning competing actions, e.g., preparing both L and R reaches in the Directional task, and then initiating the correct response following target presentation, coinciding with the Go signal. Alternatively, action planning and execution can be purely reactive, triggered by the Go signal. Either way, both strategies can be optimized through practice, leading to faster selection, planning, and initiation of actions. Independent of differences between Cued and Non-cued trials, decreases in action latency were accompanied by similarly scaled increases of perceived duration, suggesting a general link between trial-dependent improvements in response speed and changes in visual time. Additionally, the presence of the effect in the Non-cued condition rules out visuo-attentional differences between Cued and Non-cued conditions, because in the Non-cued condition the display is identical on every trial with a central, uninformative cue. Moreover, the presence of time expansion within the Non-cued condition rules out an explanation based on visuo-attentional differences between conditions. Despite differences in cue placement between Cued and Non-cued displays, in the Non-cued condition the visual display was identical on every trial, always featuring a central, uninformative cue. Nonetheless, perceived duration increased progressively across trials as participants learned to handle target location uncertainty, which involved only two possible actions and corresponding action plans, and this improvement was accompanied by a gradual reduction in action latency.

Both approaches, comparing PSEs between conditions (average responses across trials) and trial-based models relating action latency and timing response rates of change, capture the same general effect. The difference in PSEs between Cued and Non-cued reflects the overall lower latencies in the Cued condition, where the goal is clearly signaled at the beginning of the trial, allowing immediate action selection and planning. This overall advantage of Cued trials is reflected in the difference in trial-wise proportion of “longer” response intercepts between conditions (Figure S5). Reductions in action latency can have several underlying causes. These include optimized motoric processes as a function of practice and proceduralization, such as reduction in time required to select, plan, and trigger behaviors,^57^ as well as non-motoric processes, such as increase in attention,^58^ cortical arousal,^59^ and dopaminergic activity.^60^^,^^61^ Although the task itself offered no extrinsic primary rewards to reinforce reductions in response latency, engagement itself in the task might bear a time-wise opportunity cost that motivates subjects to streamline planning and action selection.^61^^,^^62^

### Action planning and neural encoding and decoding of time

Timing mechanisms are indexed by distinct neural activity patterns, reflecting transformation of sensory duration information into perceptual judgments. The accumulation of timing evidence has been linked to various time-dependent neural responses, including ramping, tuning, and speed of neural trajectories.^63^^,^^64^^,^^65^ Timekeeping is distributed across sensory, cortical, and subcortical structures, with sensory cortices locally encoding and storing temporal information embedded within sensory channels.^66^ Duration information propagates to higher order regions such as the SMA, cingulate cortex, insular cortex, BG, cerebellum, and thalamus,^6^^,^^67^^,^^68^ where it is represented in modality-independent formats that inform perceptual and decisional aspects of duration.^44^^,^^69^

Analysis of EEG data showed spreading out of Test duration-evoked responses (Figure 4A, right column), time-locked to time stimulus offset. Comparison of ERPs across tested durations showed monotonic increases in signal amplitude among linearly spaced tested durations, with amplitudes decreasing for longer durations before reaching a plateau at intermediate and longer intervals. This effect mirrors recent EEG data demonstrating distinct neural signatures of visual time, peaking at frontocentral electrodes 300–500 ms following the offset stimulus presentation.^41^^,^^70^ This evoked activity, modeled as a function of stimulus duration and participant responses, provides evidence of time decoding linked to perceptual decisions, reflecting the progressive accumulation of timing evidence toward a decision threshold. While replicating these results, our findings show that time-decoding ERP amplitudes were modulated by action conditions (Cued/Non-cued vs. Sensory; Figure 4B), with average voltage inversely related to planning specificity. This modulation is more clearly illustrated in the neurometric curves (Figure 4C), which show a shift in the voltage-duration relationship for Cued relative to Non-cued and Sensory conditions, suggesting that neural activity associated with motor preparation biases concurrent time decoding. ERP data were further examined using RSA, identifying neural activity patterns associated with perceptual distortions of time observed during action planning (relative to unplanned actions and control; Figures 5 and 6). RSA revealed that perceptual distortions of time were reflected in neural responses during time encoding, with effects selectively observed in the comparison between Cued and Non-cued action conditions, consistent with an association with motor planning. Additionally, perceptual time was reflected in neural responses during time decoding, with effects observed in both Cued vs. Non-cued and Cued vs. Sensory comparisons, suggesting a broader association with motor-related processes. This difference may reflect distinct contributions of action planning and broader motor engagement to timing-related neural activity: encoding-related activity patterns were selectively sensitive to the presence of action plans (for both Direction and Distance), while decoding-related activity patterns captured both planning-related dynamics and broader motor engagement (only in Direction). RSA also showed that timing biases were associated with neural activity in brief windows within time encoding/decoding epochs, whereas no differences were observed between conditions in prior epochs associated with processing of the goal stimulus. These results link perceptual distortions of time specifically to timing-related neural activity patterns, rather than to neural processing of non-temporal information.

Our findings align with broader evidence linking action-timing interactions with sensorimotor integration mechanisms. Functional connectivity studies demonstrate that when visual events are triggered by voluntary actions, perceptual and motor processes synchronize through oscillatory coupling across primary motor and sensory cortices.^51^ By extension, sensorimotor integration directly influences duration information carried along sensory channels, with temporal distortions explained by phase-locked sensorimotor coupling. Iwasaki et al. showed that two types of alpha rhythm modulation associated with visual stimulation and motor preparation interact in the SMA, a key region in perceptual timing, producing a shift in neural activity that can explain the subjective lengthening of perceived time.^12^ Our data add to this by showing ERP shifts that reflect action-planning specificity, i.e., action planning (captured on average by the Cued condition), as opposed to more generic motor preparation in unplanned actions (Non-cued) or disengagement of the motor system during passive observation (Sensory control) (Figure 4B). These average shifts in activity provide different neural backdrops on top of which timing computations are embedded. Different degrees of action planning establish different neural states over central electrodes, reflected in monotonic increases in average voltage across Cued, Non-cued, and Sensory conditions. Timing-evoked responses, which are sensitive to Test duration, are therefore expressed against different neural baselines across planning conditions, potentially altering the neural representation of duration indexed by these evoked responses.^41^ Our RSA analysis allowed us to link these ERP modulations to timing judgments, showing that action-planning-related shifts in the neural state were associated with the perceptual distortions observed during action planning.

Overall, our findings reveal that perceptual time is not static but is dynamically recalibrated through goal-directed action. As participants performed hand-reaching actions with varying degrees of planning specificity, perceived duration progressively expanded, driven not by execution accuracy but by gradual decreases in the timing of action selection and initiation. Trial-wise coupling between time expansion and reduced movement latency highlights the role of motor planning temporal dynamics in shaping our perceptual sense of time. Crucially, EEG/behavioral representational similarity analyses show that these distortions are associated with activity patterns during both encoding- and decoding-related periods of temporal information processing, with encoding-related effects associated with action planning and decoding-related effects with broader aspects of motor engagement and anticipation. Together, these results position action planning as an internal metric for time estimation and establish a learning-sensitive framework linking perceptual time to the evolving dynamics of goal-driven behavior. This work advances a unified perspective on how cognitive and motor systems interact to enable our perceptual experience of time.

### Limitations of the study

Given the correlational nature of the study, the relationship between changes in action latency and changes in perceived time should not be interpreted as causal. Rather, both measures may reflect common time-dependent processes accompanying performance improvements during the task, including changes in action planning through proceduralization, reward expectation, motivational engagement, or other practice-related factors. While previous work has shown that the interaction between action preparation and time expansion cannot be readily explained by non-specific attentional accounts,^16^^,^^17^ future research will be required to establish which of these factors underlies the observed relationship, and whether learning-driven changes in action planning play a primary or contributory role. A further limitation concerns the averaging approach applied to our EEG analyses. Averaging across trials allows us to characterize overall differences in neural activity associated with different levels of motor engagement and action planning but necessarily obscures trial-wise variations that may reveal learning-related activity. Future studies combining trial-level behavioral and neural analyses may help determine how perceptual and motoric performance changes over trials interact at the neural level.

## Resource availability

### Lead contact

Requests for further information and resources should be directed to and will be fulfilled by the lead contact, Nicola Binetti (nicola.binetti@uniroma2.it).

### Materials availability

No unique materials were generated in this study.

### Data and code availability

All data and code used for data analyses have been uploaded to https://osf.io/4nz2r/ and are publicly available as of the date of publication. All (raw) data reported in the manuscript (in addition to processed data currently deposited in OSF) will be shared by the lead contact upon request. Any additional information required to reanalyze the data reported in this paper is available from the lead contact upon request.

## Acknowledgments

This work was supported by a Marie Skłodowska-Curie Individual Fellowship (894955) awarded to N.B. and an ERC Consolidator grant (682117) awarded to D.B.

## Author contributions

N.B. conceived the study and constructed experiments. N.B. analyzed behavioral (timing) data. N.B. and M.Z. analyzed the EEG data. N.B. and F.M. analyzed action data and action-timing interactions. N.B., F.M., M.Z., and D.B. wrote the manuscript.

## Declaration of interests

The authors declare no conflict of interest.

## STAR★Methods

### Key resources table

**Table:** 

REAGENT or RESOURCE	SOURCE	IDENTIFIER
**Deposited data**		

Raw behavioral data	This paper	https://osf.io/4nz2r/
Processed EEG data	This paper	https://osf.io/4nz2r/

**Software and algorithms**		

MATLAB R2021a	The MathWorks	https://www.mathworks.com/; RRID: SCR_001622
Analysis code	This paper	https://osf.io/4nz2r/
Psychtoolbox	Kleiner et al.	http://psychtoolbox.org/; RRID: SCR_002881
EEGLAB v2022.1	Delorme and Makeig, 2004	https://sccn.ucsd.edu/eeglab/index.php; RRID: SCR_007292
Fieldtrip-lite v20230613 (EEGLAB plugin)	Swartz Center for Computational Neuroscience	https://eeglab.org/others/EEGLAB_Extensions.html
Python v.3.9.7	Python Software Foundation	https://www.python.org/; SCR_008394
Spyder IDE (v5.4.3)	Anaconda, Inc.	https://www.spyder-ide.org/; RRID: SCR_017585
RStudio (2023.06.1)	Posit, PBC	https://posit.co/products/open-source/rstudio/; RRID: SCR_000432
brms' package (version 2.21.0)	Stan development team	https://mc-stan.org/; RRID: SCR_023862
rstan version 2.32.6 (Stan version 2.32.2)	Stan development team	https://mc-stan.org/; RRID:SCR_018459

### Experimental model and study participant details

#### Participants

We tested 40 right-handed adults with normal or corrected-to-normal vision, in two motor-timing tasks with EEG (Directional task *N* = 20, 14 Females, 23.6 ± 2.2 years old; Distance task *N* = 20, 14 Females, 24 ± 2.9 years old). Participants were recruited through the SONA online recruitment platform, and were compensated 25€ for 2.5 h, which included hand tracking calibration, EEG cap and electrode setup while training at the action-timing task, and experimental data collection punctuated by short breaks. Informed consent was obtained by participants prior to testing. The experiment was approved by the SISSA ethics committee (prot.n. 9000 - III/13 2022) and conformed to the guidelines of the declaration of Helsinki.

#### Sample size

Sample size was informed by sample size calculations run on pilot data collected on 5 participants (dz = 0.68, target power = 0.80), consistent with numbers tested in equivalent studies in literature.^12^^,^^71^^,^^72^

### Method details

#### Apparatus

Participants performed a reaching task with their right index finger on a plexiglass surface mounted on a custom desk, while simultaneously estimating durations of brief visual stimuli. Hand position was visually occluded by the LCD monitor (DELL 1905FP, 19″, 1280x1024 pixels @ 75Hz refresh rate) positioned about 10 cm over the participant’s hand, supported by a custom wooden bracket. An infrared (IR) hand tracking camera (Leap Motion Controller - https://www.ultraleap.com/leap-motion-controller-whats-included/) captured hand movements at 75Hz (constrained to monitor refresh rate). EEG signals were recorded with an ActiveTwo EEG system (Biosemi, Amsterdam, The Netherlands) at a sampling rate of 2048 Hz with 24-bit resolution. The Biosemi amplifier applied no high-pass filter (DC-coupled) and a low-pass 5th-order sinc filter, with a −3 dB attenuation at approximately 1/5th of the selected sampling rate, resulting in an analog signal bandwidth of 417 Hz at the selected sampling frequency. The signals were acquired from 64 active electrodes mounted on an elastic cap positioned according to the standard 10/5 coordinate system. Two additional electrodes replaced the ground electrode in conventional systems: the Common Mode Sense active electrode (CMS) and the Driven Right Leg passive electrode (DRL). The signals were recorded in single-ended mode (i.e., not referenced). Four additional electrodes were used to monitor eye movements. Specifically, two electrodes were placed on the outer canthi of both eyes to record horizontal movements (hEOG-left and hEOG-right), whereas two electrodes placed beneath and above the left eye (vEOG-lower and vEOG-upper, respectively) were used to monitor vertical movements and blinks. Finally, signals from the left and right mastoids were also recorded to compute offline the average-mastoids reference. The magnitude of the offset of all electrodes was held below 20 mV. The experiment was performed in a dark and sound-proof testing booth.

#### Procedure

Participants initially performed a calibration routine to align IR camera and screen spaces by touching points with the index finger of the dominant hand on a 3x3 grid drawn on the plexiglass surface while the monitor was temporarily removed. Once calibration was completed, the monitor was secured to the bracket over the plexiglass surface. This was then followed by a training phase that allowed participants to familiarize themselves with the setup and experimental tasks, while EEG cap and electrodes were mounted. The training broke down the main components of the dual task, first focusing on reaching movements, followed by the temporal estimation task, and finally combining both tasks. In the reaching task participants had to initially position their index finger on a starting point on the plexiglass surface by aligning a small circle symbolising their index on the monitor, to a small cross in the central region of the monitor (position was randomly jittered across trials to avoid reliance on the monitor bezel as a spatial frame of reference). After reaching this position, the cross changed into an empty circle representing a fixation point lasting a variable amount of time (0.5∼1.2 s), after which a circular goal area appeared at a horizontally displaced location relative to fixation for 1 s. In the Directional task this could be at 205 pixels (approximately 5.5 cm) to the right or left of the starting point, or in the Distance task at 205 or 410 pixels to the right of the starting point. After a variable foreperiod interval ranging between 2.9 and 3.2 s, an acoustic Go signal (brief auditory beep delivered through computer speakers), instructed participants to perform a ballistic reaching movement aimed at the target location with their index finger. No online feedback on index position was provided throughout the movement, whereas position feedback was provided at the end of the reach, displaying the index’s point of arrival relative to the target area. Stimuli were displayed in gray against a black background. A score between 0 and 100 was displayed at the end of each trial above the goal area based on proximity to goal, to keep participants engaged and strive for improvement (scores were greater than 0 if the index was within the goal area, reaching 100 when the index landed on the goal area’s center). Participants were informed that at the end of the experiment they would be shown their total score (sum of trial scores), ranked based on performance relative to other tested participants. Participants were trained at the motor task until they could reliably perform ballistic reaches while waiting for the Go signal.

Participants were then trained at the timing task, without performing motor responses. Participants were first shown a fixation point (empty circle) that was intermittently filled (filled white circle), providing 15 repetitions (jittered onset) of a 0.84 s Standard duration that participants had to memorize. Then, participants were asked to compare Test durations against the memorized Standard, based on the method of single stimuli. One second following the goal presentation, the fixation point was filled (filled circle) for a brief amount of time ranging between 0.44 and 1.24 s, in 5 linearly spaced intervals, providing a Test duration. After the Go signal was presented, participants indicated with a left or right mouse button press (held with their left, non-reaching hand), whether the Test appeared shorter or longer than the Standard duration (left or right button, respectively). Finally participants were trained with combined motor and timing tasks, thus requiring them to monitor the Test stimulus as they prepared the reaching action, and to judge its duration after the completion of the action. Participants were trained until they reached 100% accuracy at the timing task with the shortest and longest Test durations (0.44 & 1.24 s Tests).

The testing phase involved 3 conditions (counterbalanced across participants), where time was estimated while: 1) planning hand movements aimed at cued goal locations (Cued condition - priming a specific plan initiated at the Go signal, as done in training), 2) during the foreperiod of reactive movements aimed at Non-cued goals (Non-cued condition - a non-informative goal is shown on top of the starting point, requiring a selection amongst alternative action plans at the Go signal), and 3) during matched intervals involving no reaching movements (Sensory). The Cued condition involved 20 blocks of 20 trials each. Each Cued block consisted in congruent or incongruent trials, where we manipulated camera to screen-space mappings: e.g., in the Directional task a leftward hand movement produces a leftward (congruent) or rightward (incongruent) movement on screen space, or in the Distance task a small hand movement produces a small (congruent) or large (incongruent) displacement in screen space. Congruent and Incongruent blocks were interleaved (i.e., alternated every 20 trials), with a text prompt informing participants on the current block type. The Block order (starting with Congruent and Incongruent) was counterbalanced across participants. Thus Cued condition involved four types of trial for Directional task (Left Congruent - LC; Right Congruent - RC; Left Incongruent - LI; Reft Incongruent- RI) and for the Distance task (Near Congruent -NC; Far Congruent - FC; Near Incongruent - NI; Far Incongruent - FI). These manipulations were aimed at studying how planned action features and their predicted sensory consequences (which can be disentangled based on Congruent and Incongruent mappings), affect timing estimates. Non-cued and Sensory conditions were alternated in 5 blocks of 20 trials each (i.e., 20 Non-cued, 20 Sensory, 20 Non-cued etc). Cued Vs. Non-cued/Sensory condition order was counterbalanced across participants.

Stimulus presentation and response collection were controlled through psychtoolbox 3^73^ running on MATLAB R2021a.^74^

#### EEG recording

• EEG signals were recorded with an ActiveTwo EEG system (Biosemi, Amsterdam, The Netherlands) at a sampling rate of 2048 Hz and a 24-bit resolution. The Biosemi amplifier applied no high-pass filter (DC-coupled) and a low-pass 5th-order sinc filter, with a −3 dB attenuation at approximately 1/5th of the selected sample rate, resulting in an analog signal bandwidth of 417 Hz at the selected sampling frequency.
• The signals were acquired from 64 active electrodes mounted on an elastic cap and positioned according to the standard 10/10 coordinate system. Two additional electrodes replaced the ground electrode in conventional systems: the Common Mode Sense active electrode (CMS) and the Driven Right Leg passive electrode (DRL). The signals were recorded in single-ended mode (i.e., not referenced). Four additional electrodes were used to monitor eye movements. Specifically, two electrodes were placed on the outer canthi of both eyes to record horizontal movements (hEOG-left and hEOG-right), whereas two electrodes placed beneath and above the left eye (vEOG-lower and vEOG-upper, respectively) were used to monitor vertical movements and blinks. Finally, the signals from the left and right mastoids were also recorded to compute offline the average-mastoids reference.
• The magnitude of the offset of all electrodes was held below 20 mV.

#### EEG pre-processing

• EEG signals were processed offline using custom scripts and the EEGLAB toolbox (v2022.1)^75^ in the MATLAB environment (R2021a).^74^
• Data were imported in EEGLAB, re-referenced to the average of the signals recorded from left and right mastoids, resampled to 512 Hz and filtered, by applying a Hamming windowed sinc FIR filter, bandpass edges 0.5–40 Hz (using the function *pop_eegfiltnew* for EEGLAB, in the plugin “firfilt” v2.7.1).
• EEG signals were visually inspected to identify and remove bad channels.
• Blinks and eye-movement artifacts were corrected with a procedure based on Independent Component Analysis (ICA). IC weights were calculated on a separate dataset, created from the original dataset by applying a bandpass filter (1–30 Hz), re-sampling to 128 Hz. The FastICA algorithm (function *fastica,* version 2.5; http://www.cis.hut.fi/projects/ica/fastica/) was used and IC weights were transferred to the original dataset.
• ICs were automatically labeled with the *pop_iclabel* function for EEGLAB, in the “ICLabel” plugin.^76^ Additionally, ICs accounting for blinks and/or eye movements were marked with the following procedure. (i) IC waveforms were correlated (Spearman correlation) with EOG signals. (ii) The most correlated IC with hEOG (computed as hEOG-left minus hEOG-right) was marked as IC accounting for horizontal eye movements. (iii) The most correlated IC with vEOG (computed as vEOG-upper minus vEOG-lower) was marked as IC accounting for blinks and vertical eye movements. ICs were visually inspected to confirm the selection based on the automatic labeling and the correlation with EOG channels, and removed.
• EEG epochs were extracted to compute event-related potentials (ERPs) for the encoding and the decoding epochs. Encoding epochs were extracted to assess whether action planning set a condition-specific “state” for time accumulation, before any information about Test duration is provided (e.g.,: before the offset of the shortest probe). The time-locking event was the Test onset, the interval was from −200 ms to 434 ms (i.e., the shortest time interval before the Test offset) and the interval considered for baseline correction was from −200 ms to 0 ms. A voltage-based threshold of ±100 μV was applied for bad epoch rejection. Decoding epochs were extracted to assess whether action planning affects readout of previously encoded Test duration. The time-locking event was the Test offset, the interval was from −50 ms to 617 ms (i.e., the shortest time interval before the go signal) and the interval considered for baseline correction was from −50 ms to 50 ms.^41^ A voltage-based threshold of ±100 μV was applied for bad epoch rejection. We additionally monitored neural activity associated to the processing of the goal stimulus, time locked to presentation of Cued informative/Non-cued non-informative cue stimulus (0.5 s).
• A third set of epochs was also extracted for supplementary analyses. The time-locking event was the Test onset, the interval was from −200 ms to 2000 ms (i.e., encompassing both the encoding and the decoding) and the interval considered for baseline correction was from −200 ms to 0 ms. A voltage-based threshold of ±100 μV was applied for bad epoch rejection.

#### Extraction of action performance features

We extracted action features (SI) from raw hand positional data in order to characterize reaching action performance and relate performance measures to timing estimates throughout trials. We parsed hand positional data to identify time at which each ballistic action started and terminated, based on a fixed velocity threshold. We defined ‘Action Latency’ as the time required to initiate each reach, therefore providing a measure of action readiness. We calculated ‘Mean Velocity’ of hand movements during hand displacement (i.e., mean velocity during interval between the start and end of each reach). We calculated ‘Reaching Error’ as the difference between the final screen position occupied by the hand at the end of each reach and the target, providing a measure of reaching precision. We extracted ‘Distance Traced’ as the product of action duration (interval between start and end of each reach) and mean velocity, indicating the amount of space covered by the reach.

### Quantification and statistical analysis

#### EEG analysis

• ERPs for Encoding and Decoding were analyzed separately for Direction and Distance tasks. Mass-univariate permutation-based tests, with a cluster-based method for multiple comparison correction, were implemented. Specifically, the analyses aimed at identify time points and cluster of electrodes that showed a significant effect of the Test duration (i.e., when and where ERP amplitudes differentiated among the 5 Test durations) or a significant effect of condition (i.e., when and where ERP amplitudes differentiated among Cued/Non-cued/Sensory).
• Tests were run with the function *ft_timelockstatistics* for EEGLAB, in the “Fieldtrip-lite” plugin (v20230613). Number of permutations was set to 1000, p-values for both cluster threshold and significance threshold was set to 0.01.

#### Behavioral models

• *Timing judgment and Action condition modeling.* Timing perceptual judgements (proportion of “Test longer” responses) as a function of log Test durations, were fit with a Logistic function, allowing the extraction of the Point of Subjective Equality (PSE - the midpoint of the psychometric curve, identifying the Test duration that is perceptually matched to the Standard) and Just Noticeable Difference (JND - mean difference in Test duration yielding 0.25 and 0.75 proportion of “Test longer” responses, quantifying perceptual sensitivity to differences in Test durations). Linear Mixed Effects Model (LME) was used to study differences in PSE and JND, with Condition (Cued = LC, RC, LI, RI, Non-Cued and Control for Directional task; Cued = NC,FC,NI,FI, Non-Cued and Control for Distance task) as factor and participants as random effects (PSE ∼ Condition + (1 | subID); JND ∼ Condition + (1 | subID)). Data were analyzed with the Spyder IDE (v5.4.3) running Python 3.9.7.
• *Effects of experimental manipulations and action performance features on timing.* The experimental paradigm was designed to study effects of motor preparation and plan selection on timing computations, as well as test how planned motor parameters (Directional or Distance features, congruently or incongruently mapped onto screen space) impact on timing. These questions were addressed through control of specific experimental variables. By contrast, the questions regarding the role of action performance measures on timing involve variables that were not directly controlled in the experimental design. The first concerned whether any of our performance variables (such as action latencies or the distance traveled while reaching the goal) might ultimately influence time perception. The second asks whether any such performance measure might explain the particular evolution of duration judgements across the duration of a block, or the entire task — reflecting an influence of learning, variations in engagement, vigor other factors on timing.

To examine the relationship between motor variables and timing judgments, we constructed a comprehensive model using the BRMS package.^77^ The model included the following variables: (1) Test durations, modeled non-parametrically with one free parameter per duration; (2) log-transformed action latencies; (3) a factor indicating the task being performed; (4) the line-projected distance traveled to reach the target; (5) the average movement speed throughout the reach; and (6) the precision, defined as the square root of the final distance to the target. These variables, with the addition of an intercept, interacted additively and were then logit-transformed to estimate the likelihood of the temporal judgment. The model can be run in R and is available on OSF at https://osf.io/4nz2r/.

• *Changes in time perception across trials*. To assess changes in log-latencies and duration judgements across trials, we developed an additional Bayesian regression model using the BRMS package. This model included fixed effects for Test durations and normalized trial numbers – both within each block and across the entire cued/non-cued condition. Crucially, individual differences in the slopes of these effects were modeled as random effects. This allowed us to estimate, for each subject, their rate of change (acceleration or deceleration) in response latency and in perceived time (expansion or compression), which we could then relate statistically. For parsimony in hypothesis testing, we conflated rates of change in the Cued and Non-cued conditions (i.e., we considered these conditions as a single condition) into a single parameter (random-effect) per-subject. For log-latencies, the likelihood distribution used was Gaussian; for temporal judgements, it was a standard logistic (or inverse logit) function. The model formulas were:
  $$\left[temporal\;j./\log\left(latency\right)\right]\;∼\;1+\underline{p}+t_{c,b}+t_{c}+\left(1+\underline{p}+t_{c,b}+t_{c}+|i\right)$$

p is a vector indicating all Test durations (each modeled with its own coefficient to exclude probe-specific effects non-parametrically). *t*_*c*,*b*_ is the scaled (divided by the number of last trial, e.g., 20) trial number within each block and condition. *t*_*c*_ is the scaled (again divided by the number of last trial; Cued: 200, Non-Cued: 400) trial number within each condition. The part in parentheses represent participant-specific random slopes associated with the across-trial effect in separate multilevel models for log-action latency and timing responses, estimated after controlling for Test duration and other fixed effects, which were used for post-hoc correlations, consistent with BRMS notation (*i* indicates subject number).

Trial progression was defined within each condition (Cued/Non-cued – the sensory conditions does not pertain to this analysis because action was not required in that condition). So the slope captures change over time inside that condition only. Because the predictor is condition-specific, it is not indexing overall experiment time and therefore is not confounded with whether that condition occurred first or second.

Given sufficient inter-individual variability, this approach enables the investigation of the relationship between the rates of change in duration judgements and latencies (alpha parameters that capture a linear rate of change in timing estimates and in action latency over and above the fixed effects). Spearman correlation coefficients were used to evaluate these correlations.

• *Learning differences between experimental conditions.* To examine whether trial-by-trial learning trajectories differed between cue conditions, we fit separate mixed-effects models for the cued and non-cued conditions. Analyses were performed on trials from the experimental and motor-control conditions only, excluding sensory-control trials. We applied the same trial-level filtering used in the main analyses. We included two temporal predictors: normalized trial number within condition, indexing gradual changes across the condition, and normalized trial number within block, indexing local within-block changes. Both predictors ranged from 0 to 1 within the relevant grouping.

For temporal judgments, we fit a logistic mixed-effects model because the response was binary:

$$logit\left(P\left(y_{ij}=1\right)\right)=\beta_{0}+\beta_{1}\;ProbeDur_{ij}+\beta_{2}\;Task_{ij}+\beta_{3}\;TrialCond_{ij}+\beta_{4}\;TrialBlock_{ij}+u_{0j}+u_{3j}\;TrialCond_{ij}+u_{4j}\;TrialBlock_{ij}$$

Here, *y*_*ij*_ indicates whether participant j gave a “longer” response on trial i, TrialCond is normalized trial number within condition, and TrialBlock is normalized trial number within block. For action-start latencies, we fit an analogous linear mixed-effects model to log-transformed action-start latency:

$$\log\left(ActStart_{ij}\right))=\beta_{0}+\beta_{1}\;ProbeDur_{ij}+\beta_{2}\;Task_{ij}+\beta_{3}\;TrialCond_{ij}+\beta_{4}\;TrialBlock_{ij}+u_{0j}+u_{3j}\;TrialCond_{ij}+u_{4j}\;TrialBlock_{ij}+\epsilon_{ij}$$

Both equations include fixed effects *β* that estimate average relationships across participants and random effects *u*that allow the intercept and selected slopes to vary by participant (j).

The logistic model predicts a binary outcome on the log-odds scale, whereas the lin ear model predicts a log-transformed continuous outcome and includes a residual error term (e.g., *ϵ*_*ij*_).

Random effects were estimated by participant and included random intercepts and random slopes for both trial-number predictors. Models were fit separately for the cued and non-cued conditions.

In this analysis separating the two conditions, we found that effects were stronger in the non-cued condition. For time responses, the trial effect was positive in both conditions, but weaker (non-significant) in the cued condition, b = 0.37, SE = 0.21, z = 1.72, *p* = 0.086, and reliable in the non-cued condition, b = 0.67, SE = 0.19, z = 3.48, *p* < 0.001. For action-start latencies, the decrease was weak in the cued condition, b = −0.02, SE = 0.03, t = −0.87, but clear in the non-cued condition, b = −0.14, SE = 0.02, t = −5.67. We also examined whether individual differences in learning slopes for time judgements were associated with individual differences in action-start latency slopes, separately for each cue condition. In the cued condition, the subject-level across-condition slopes were negatively correlated, r = −0.31, 95% CI [-0.45, −0.16], indicating that participants who showed stronger increases in “longer” responses also tended to show stronger decreases in action-start latency. This association was weaker and uncertain in the non-cued condition, r = −0.11, 95% CI [-0.42, 0.22]. Within-block slope correlations were close to zero in both conditions.

• *Action performance effects on subsequent temporal judgements.* To test whether temporal judgments were influenced by action performance on the preceding trial, we fit exploratory Bayesian mixed-effects models predicting the current temporal judgment from lagged performance measures. Analyses were restricted to experimental and motor-control trials, excluding sensory-control trials because action-performance measures were unavailable in that condition. We applied the same trial-level filtering as in the main analyses, retaining trials with valid goal distances below 200 and action-start latencies between 11 and 75 samples. For each participant and task, we identified the immediately preceding trial. To avoid carrying information across block boundaries, the primary lagged analysis retained only trials for which the preceding trial occurred within the same block; first trials within each block were therefore excluded. We examined two lagged predictors in separate models: absolute hand error on the preceding trial and score on the preceding trial. Both predictors were z-scored within participant.

Because temporal judgments were binary, models used a Bernoulli likelihood with a logit link. For the lagged absolute-error model, the equation was:

$$logit\left(P\left(y_{ij}=1\right)\right)=\beta_{0}+\beta_{1}\;ProbeDur_{ij}+\beta_{2}\;Task_{ij}+\beta_{3}\;Condition_{ij}+\beta_{4}\;TrialCond_{ij}+\beta_{5}\;TrialBlock_{ij}+\beta_{6}\;LagError_{ij}+\beta_{7}\;LagError_{ij}\times Condition_{ij}+u_{0j}\text{.}$$

For the lagged-score model, the model was the same, except that the LagError term was replaced by a LagScore term. In these models, *y*_*ij*_ indicates whether participant j responded “longer” on trial i, TrialCond is normalized trial number within condition, and TrialBlock is normalized trial number within block. Condition coded the cued versus non-cued condition. The participant-level *u*_0*j*_ term denotes a random intercept. Models were fit separately for previous-trial absolute hand error and previous-trial score, allowing the lagged effect to differ between cue conditions through the interaction with condition.

Previous-trial score did not reliably predict current temporal judgments in either the cued condition, b = −0.02, 95% CrI [-0.08, 0.04], or the non-cued condition, b = 0.04, 95% CrI [-0.09, 0.18]. For previous-trial absolute hand error, the effect was uncertain in the cued condition, b = −0.01, 95% CrI [-0.07, 0.04], but there was evidence for a negative association in the non-cued condition, b = −0.16, 95% CrI [-0.30, −0.02]. This suggests that, in the non-cued condition, larger action errors on the preceding trial were associated with a lower probability of responding “longer” on the current trial. We now report this exploratory lagged analysis in the Supplementary Information. Importantly, this effect was condition-specific and was not observed for the score-based performance measure, so we interpret it cautiously. The results suggest that prior-trial action error may have some local influence on temporal judgments in the non-cued condition, but they do not indicate that prior-trial feedback explains the broader trial-by-trial evolution of temporal perception.

#### Neural correlates of perceptual timing bias induced by action preparation

We identified the neural source of perceptual distortions (bias) of time occurring throughout the experiment using Representational Similarity Analysis^78^ (RSA), a computational technique that allows identifying correlates of behavioral processes based on similarly patterned EEG responses. For each participant, this involved calculating timing judgment Representational Dissimilarity Matrices (Behavioral RDMs), accounting for differences (distances in Euclidean space) in proportion of “Test longer” responses across all conditions (Cued, Non-cued and Control) and Test durations (Figure 5). Information structure within these matrices captures each participant’s overall performance at the timing task. This pattern reflects differences in timing judgements related to Test duration, and biases related to experimental condition. The perceptual bias in the Cued condition is reflected in asymmetries in Euclidean distances between the upper and lower triangle regions in RDM quadrants contrasting Cued against Non-cued and Sensory conditions (Figure 5A, Behavioral RDM). Neural activity was represented in an equivalent format (EEG RDMs), where for each EEG channel and 50 ms time window spanning the time Encoding (440 ms following Test onset) and Decoding epochs (620 ms following Test offset) belonging to each participant, we calculated differences in neural response (Euclidean distance in average voltage) across conditions and Test durations. Behavioral and EEG RDMs were compared via Kendall correlations to identify - where (which channels) and when (at what time windows) - the representational structure of neural activity resembled that of timing performance. Given that we specifically aimed at identifying the neural source of biases in perceptual time, we contrasted RDM correlations against a null model hypothesis, representing unbiased time perception. This rationale provided means of identifying channels/time windows of EEG activity that reflect biased estimates of duration, based on how they differ relative to unbiased time perception. This was achieved by generating “theoretical” timing judgment RDMs for each participant (Unbiased-Behavioural RDMs), removing timing biases within Cued, Non-cued and Control conditions. Participants’ psychometric fits were shifted so their midpoints coincided with the Standard duration (i.e., corresponding to an unbiased PSE), and proportion of “Test longer” responses were estimated across Test durations based on fit parameters, thus preserving sensitivity information reflected in curve slopes. This yielded RDMs with symmetrical Euclidean distances between upper and lower triangle regions within RDM quadrants comparing different experimental conditions (Figure 5, Behavioral RDM - no-bais). We computed Euclidean distance because the behavioral data are bounded and non-linear, and ED is less prone to the bias introduced by correlation-based metrics that assume unbounded variables. For consistency, we applied the same metric to EEG values. Each condition–duration pair is represented by a single summary value, and RDMs therefore represent differences in response magnitude rather than multivariate patterns. Behavioral/EEG RDM r-scores and Unbiased-Behavioural/EEG RDM Kendall coefficients were then converted to z-scores (Fisher transform), and compared via t-tests (FDR corrected p-scores across 64 channels), yielding t-score heatmaps contrasting biased and unbiased time perception (Figures 5B and 5C).
