# Supplementary material for: Perceptual lengthening of visual time throughout learning of hand-reaching actions
Source: iScience. 2026 Oct 25;29(11):117993. doi: 10.1016/j.isci.2026.117993 (PMC13722560; doi:10.1016/j.isci.2026.117993)
Supplement: Supplementary file 1 [file mmc1.pdf]

iScience, Volume *29*

Supplemental information

Perceptual lengthening of visual time throughout learning of hand-reaching actions

Nicola Binetti, Federico Mancinelli, Marco Zanon, and Domenica Bueti

Perceptual lengthening of visual time throughout learning of hand-reaching actions - Supplemental Information

Nicola Binetti*[1,2], Federico Mancinelli[1,3], Marco Zanon[1] Domenica Bueti[*1]

1. International School for Advanced Studies (SISSA), Trieste, Italy

2. Dipartimento di Medicina dei Sistemi, Università degli Studi di Roma Tor Vergata, Rome, Italy

3. University of Bonn, Bonn, Germany

* dual senior authorship

Corresponding author: nicola.binetti@uniroma2.it

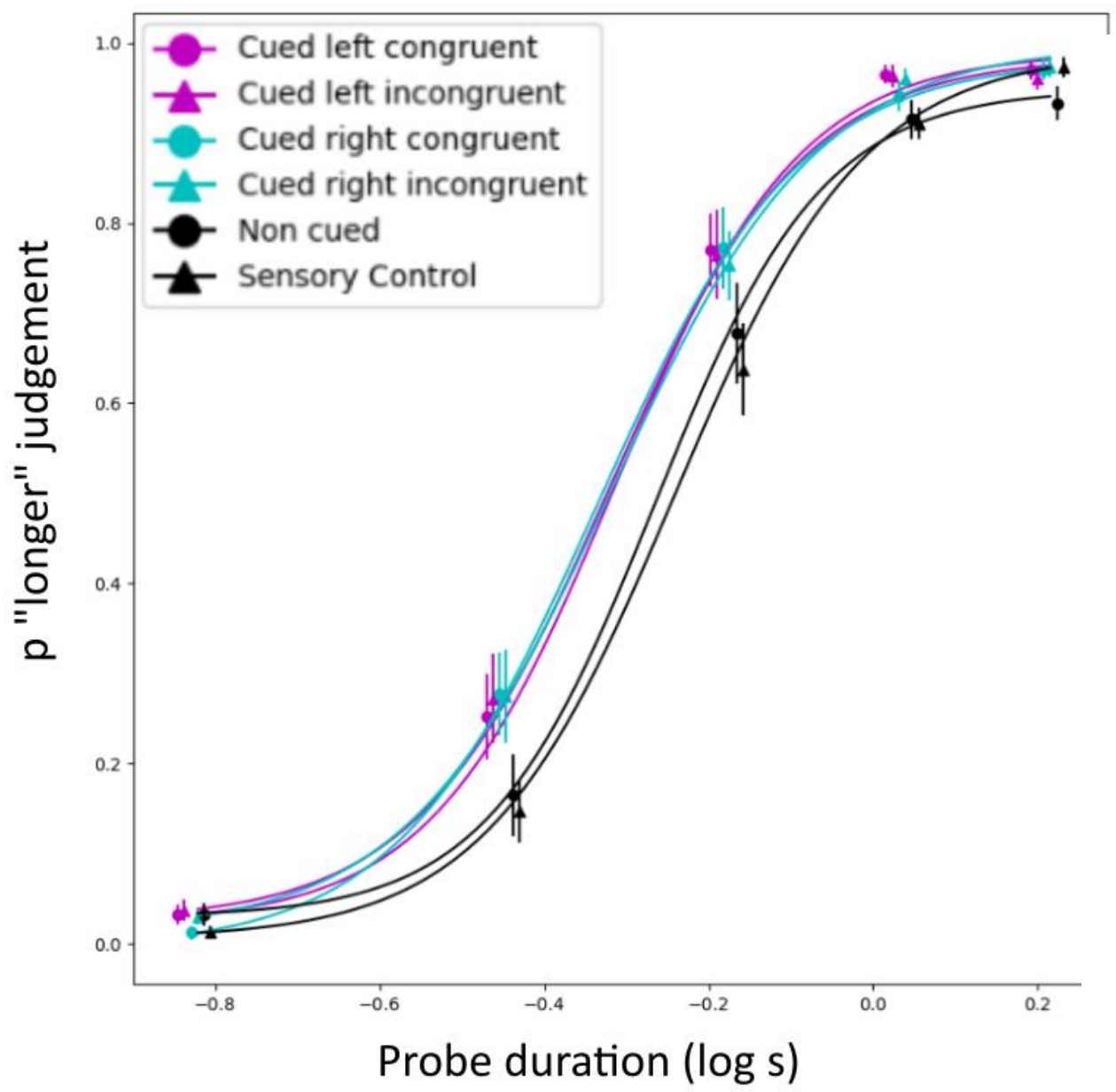

Directional task contrasts:

contrast	estimate	SE	df	t.ratio	p.value
LC - LI	-0.001875	0.0133	95	-0.141	1.0000
LC - NonCued	-0.038807	0.0133	95	-2.910	0.0498
LC - RC	0.000199	0.0133	95	0.015	1.0000
LC - RI	0.003784	0.0133	95	0.284	0.9997
LC - Sensory	-0.053774	0.0133	95	-4.033	0.0015
LI - NonCued	-0.036932	0.0133	95	-2.770	0.0713
LI - RC	0.002074	0.0133	95	0.156	1.0000
LI - RI	0.005659	0.0133	95	0.424	0.9982
LI - Sensory	-0.051899	0.0133	95	-3.892	0.0025
NonCued - RC	0.039006	0.0133	95	2.925	0.0478
NonCued - RI	0.042591	0.0133	95	3.194	0.0227
NonCued - Sensory	-0.014967	0.0133	95	-1.123	0.8709
RC - RI	0.003585	0.0133	95	0.269	0.9998
RC - Sensory	-0.053973	0.0133	95	-4.048	0.0014
RI - Sensory	-0.057558	0.0133	95	-4.317	0.0005

Figure S1: Psychometric fit and PSE (point of subjective equality) LME ANOVA contrasts of 6 conditions (4 Cued, Non-cued and Sensory control) - Direction task. Cued trials (target is shown during foreperiod) are split in 4 types based on action type (left or right hand reach) and hand to screen space mapping (congruent or incongruent): LC = Left Congruent; RC = Right Congruent; LI = Left Incongruent (right hand movement to reach Left target); RI = Reft Incongruent (left hand movement to reach Right target). NonCued (target is not shown during foreperiod, and its presentation coincides with the Go signal). Sensory (Test is timed in matched interval where action is not prepared). Error bars represent standard error of the mean (s.e.m).

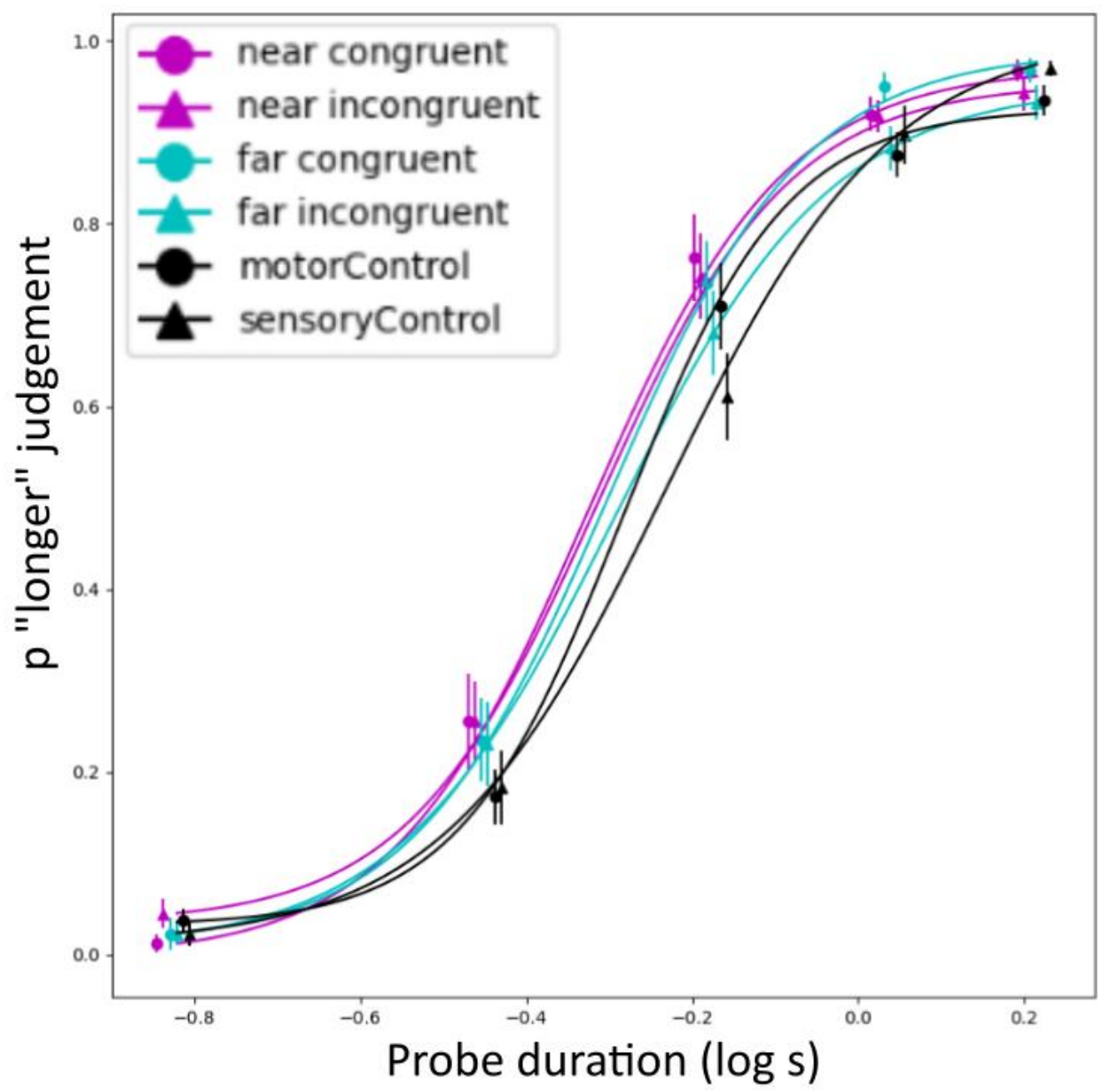

Distance task contrasts:

contrast	estimate	SE	df	t.ratio	p.value
FC - FI	-0.00788	0.0173	95	-0.457	0.9974
FC - NC	-0.00990	0.0173	95	-0.574	0.9925
FC - NI	-0.00419	0.0173	95	-0.243	0.9999
FC - NonCued	-0.02829	0.0173	95	-1.639	0.5748
FC - Sensory	-0.05745	0.0173	95	-3.330	0.0152
FI - NC	-0.00202	0.0173	95	-0.117	1.0000
FI - NI	0.00369	0.0173	95	0.214	0.9999
FI - NonCued	-0.02041	0.0173	95	-1.183	0.8440
FI - Sensory	-0.04958	0.0173	95	-2.873	0.0548
NC - NI	0.00570	0.0173	95	0.331	0.9995
NC - NonCued	-0.01839	0.0173	95	-1.066	0.8936
NC - Sensory	-0.04756	0.0173	95	-2.756	0.0738
NI - NonCued	-0.02409	0.0173	95	-1.396	0.7291
NI - Sensory	-0.05326	0.0173	95	-3.087	0.0308
NonCued - Sensory	-0.02917	0.0173	95	-1.691	0.5414

Figure S2: Psychometric fit and PSE (point of subjective equality) LME ANOVA contrasts of 6 conditions (4 Cued, Non-cued and Sensory control) - Distance task. Cued trials (target is shown during foreperiod) are split in 4 types based on action type (rightward near or far hand reach) and hand to screen space mapping (congruent or incongruent): NC = Near Congruent; FC = Far Congruent; NI = Near Incongruent (large hand movement to reach Near target); FI = Far Incongruent (small movement to reach Far target). Error bars represent standard error of the mean (s.e.m).

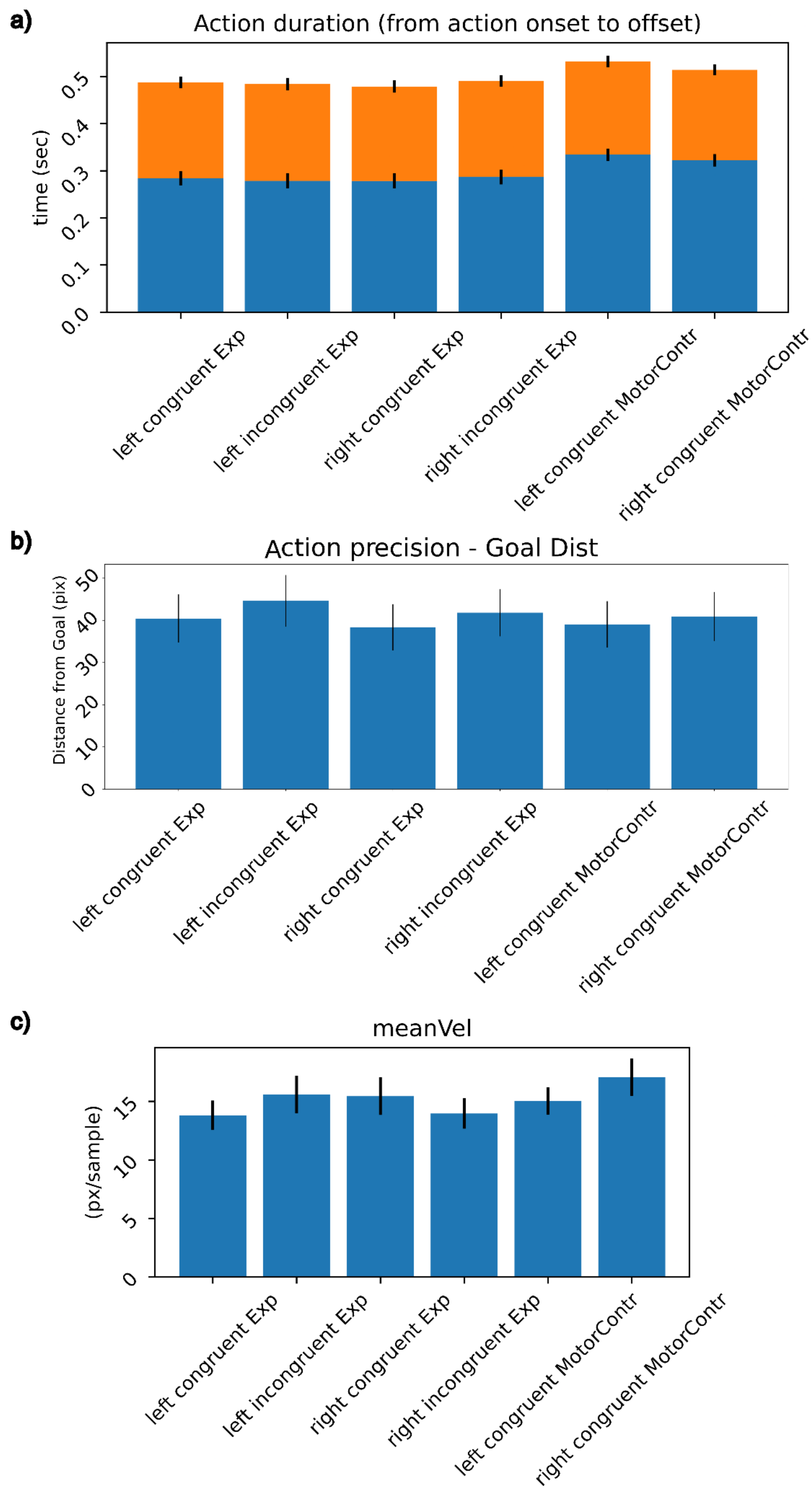

Figure S3: Direction task action features across 6 experimental conditions. a) Action latency (blue) and duration (orange), respectively measured as interval between go signal and action start, and interval between action start and action end. b) Reaching errors across conditions, measured as distance between landing point and target center. c) Mean action velocity. Error bars represent standard error of the mean (s.e.m).

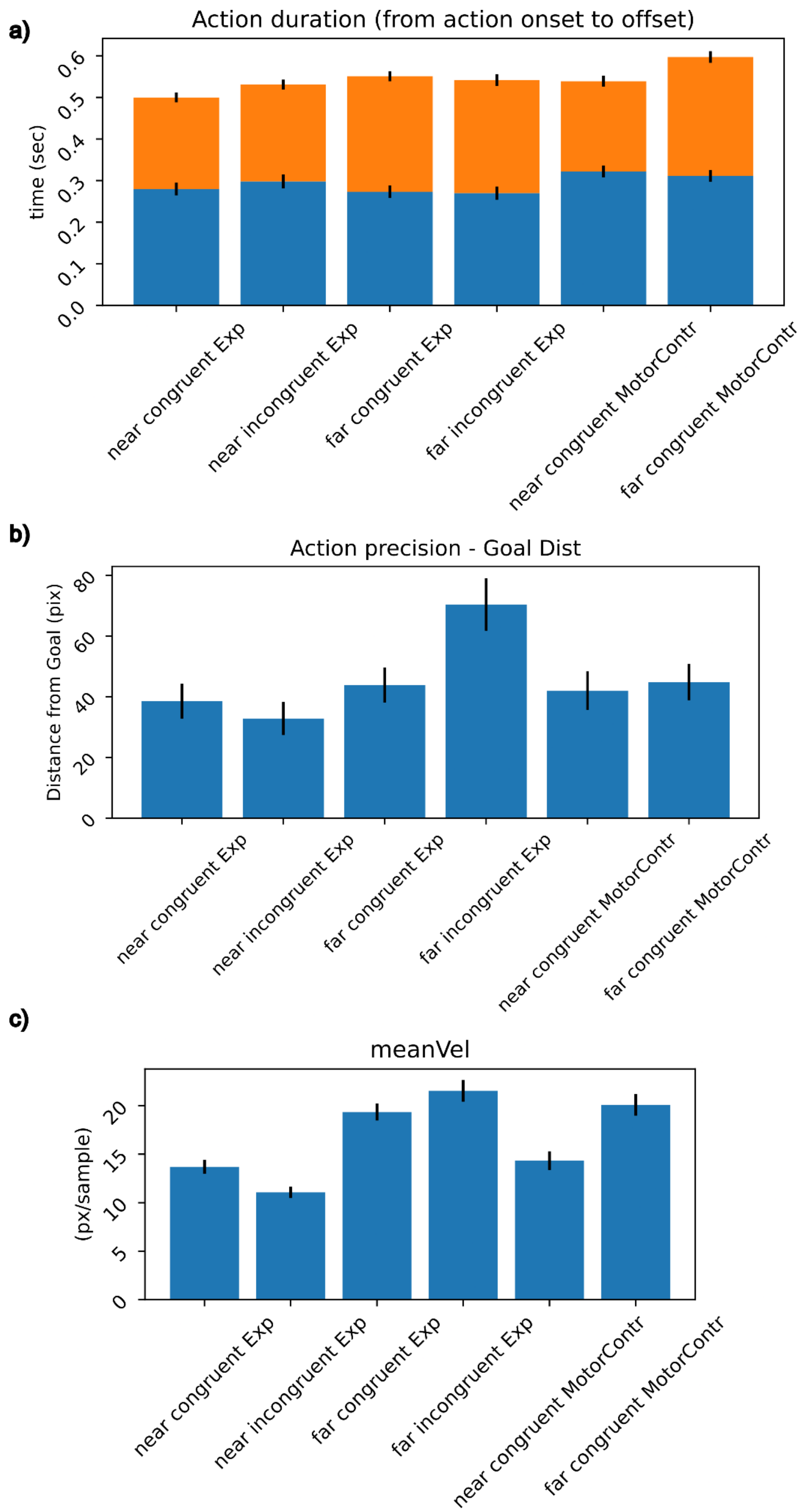

Figure S4: Distance task action features across 6 experimental conditions. a) Action latency (blue) and duration (orange). b) Reaching errors across conditions, measured as distance between landing point and target center. c) Mean action velocity. Error bars represent standard error of the mean (s.e.m).

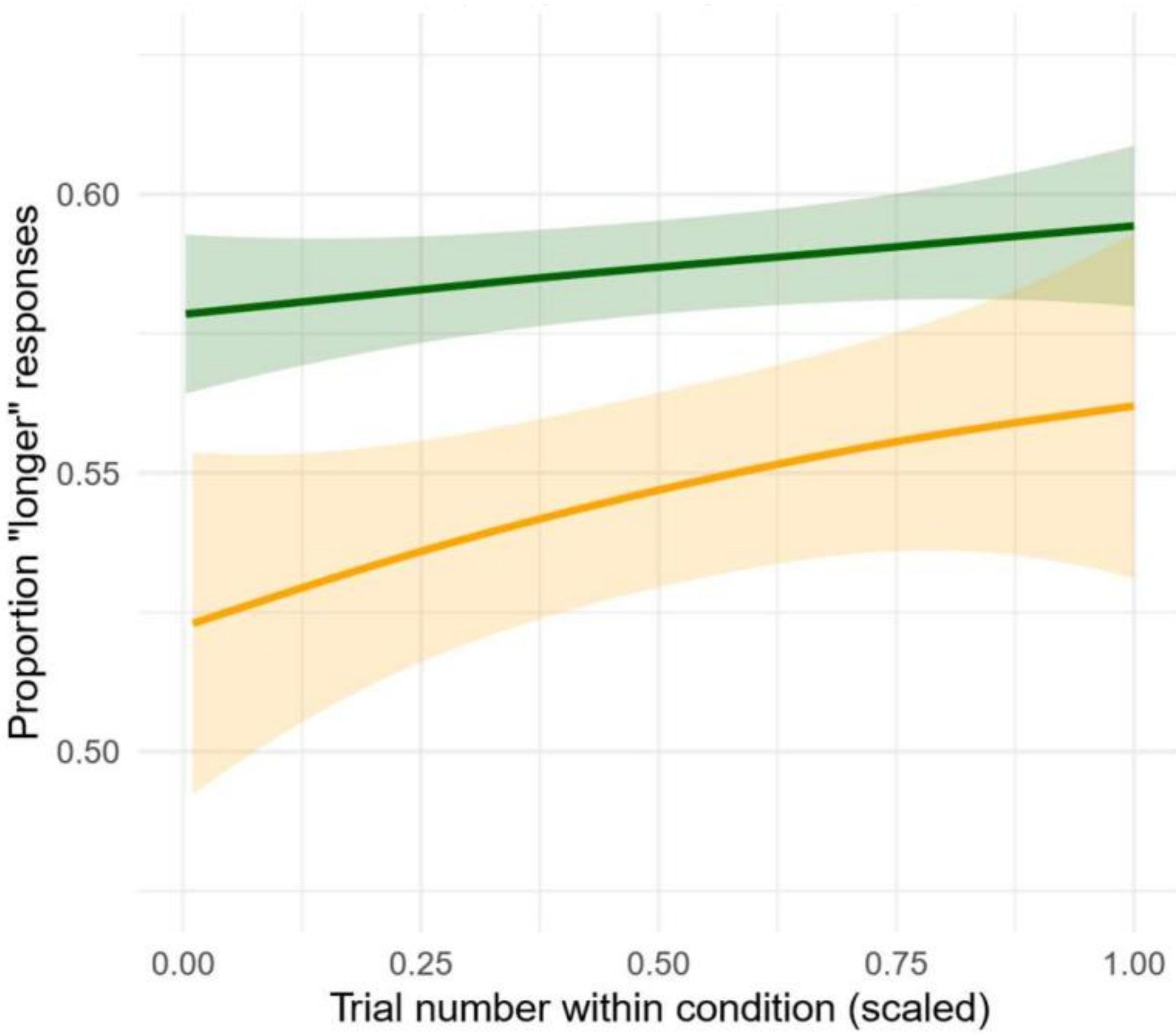

Figure S5: Timing judgements as a function of trial number, showing progressive expansion of perceived time. Rates of change in timing judgements were individually modelled for the Cued (green) and Non-cued (orange) conditions. The differences in intercept between these curves reflect the overall difference in PSEs estimating through psychometric fits. Shaded Error bars represent standard error of the mean (s.e.m).

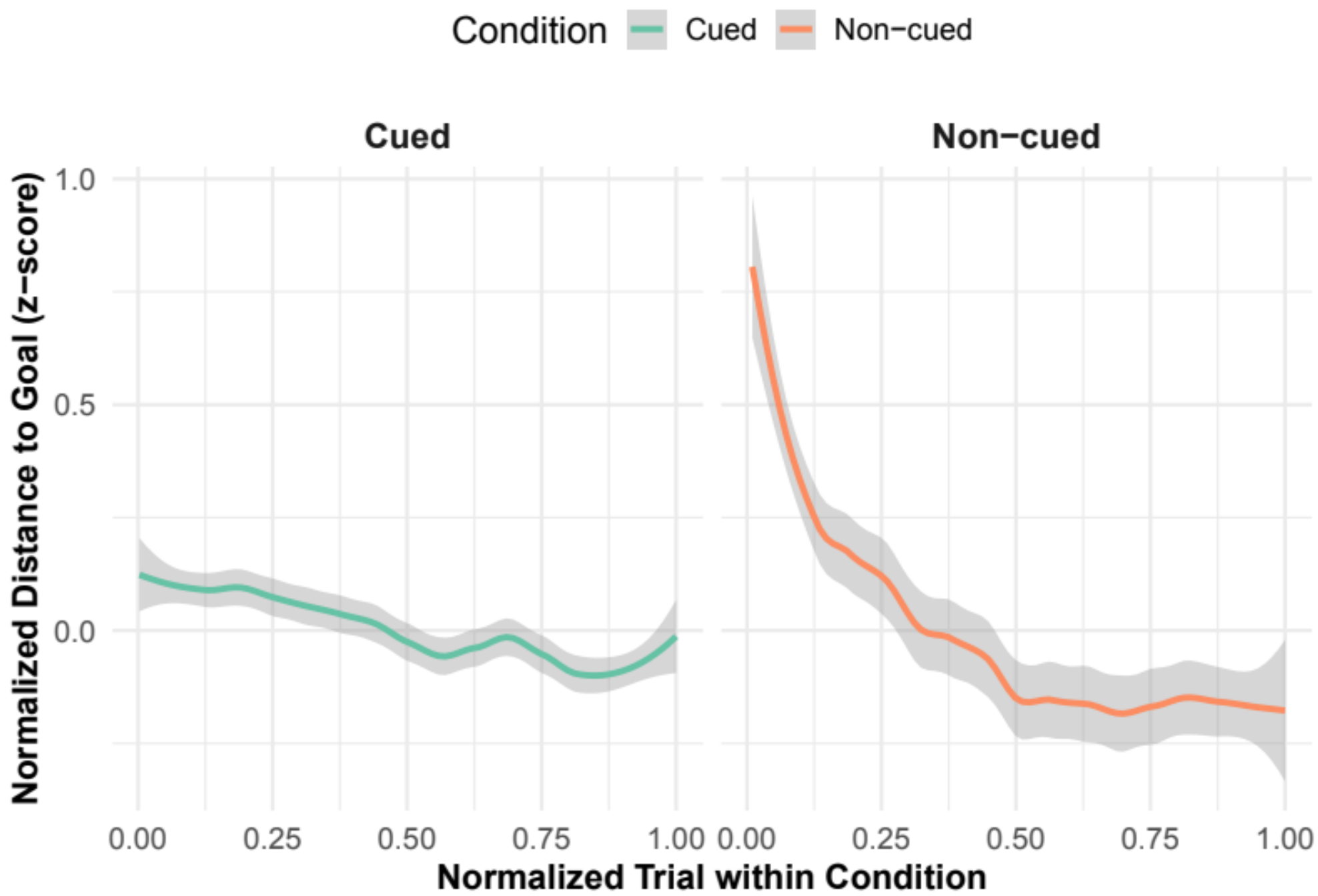

Figure S6: Hand reach precision (normalized distance from goal area centre) as a function of trial number, showing progressive improvement in action precision. Rates of change in timing judgements were individually modelled for the Cued (green) and Non-cued (orange) conditions. Rates of change in precision are more pronounced in Non-cued trials, reflecting additional cost in handling target location uncertainty. Shaded error bars represent standard error of the mean (s.e.m).

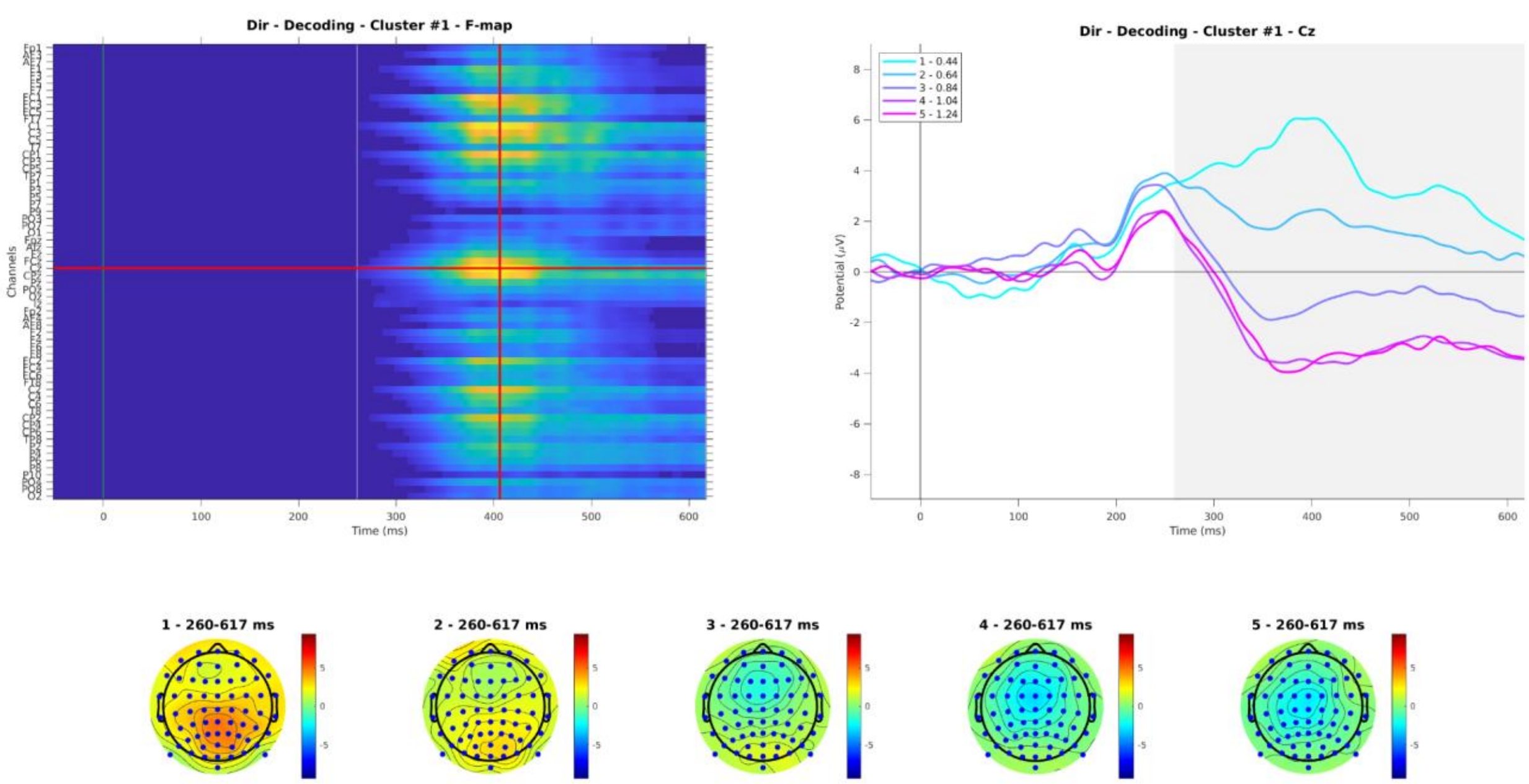

Figure S7: Direction Task Time Decoding ERP analysis - Effect of Probe at electrode Cz

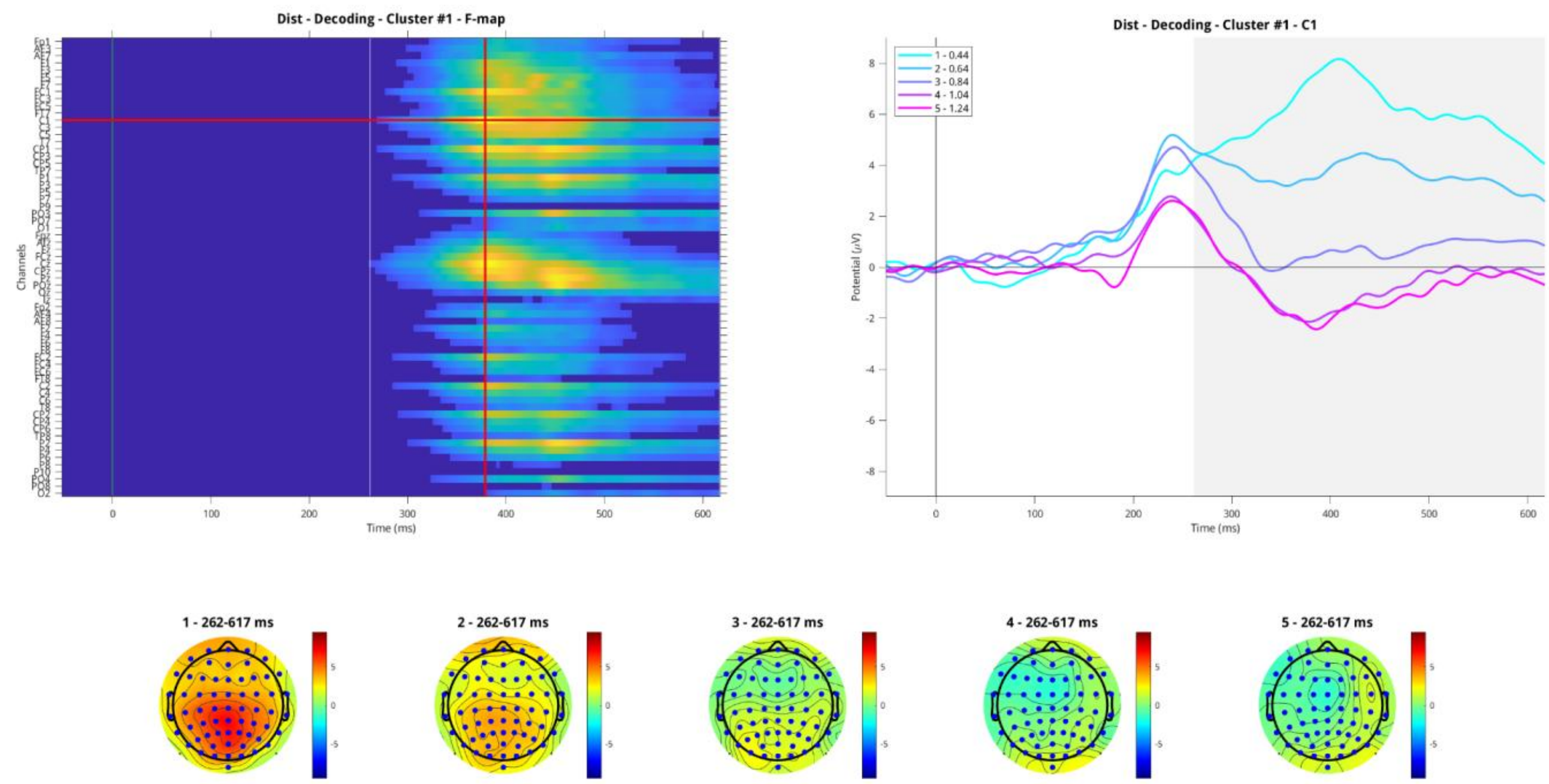

Figure S8: Distance Task Time Decoding ERP analysis - Effect of Probe at electrode C1

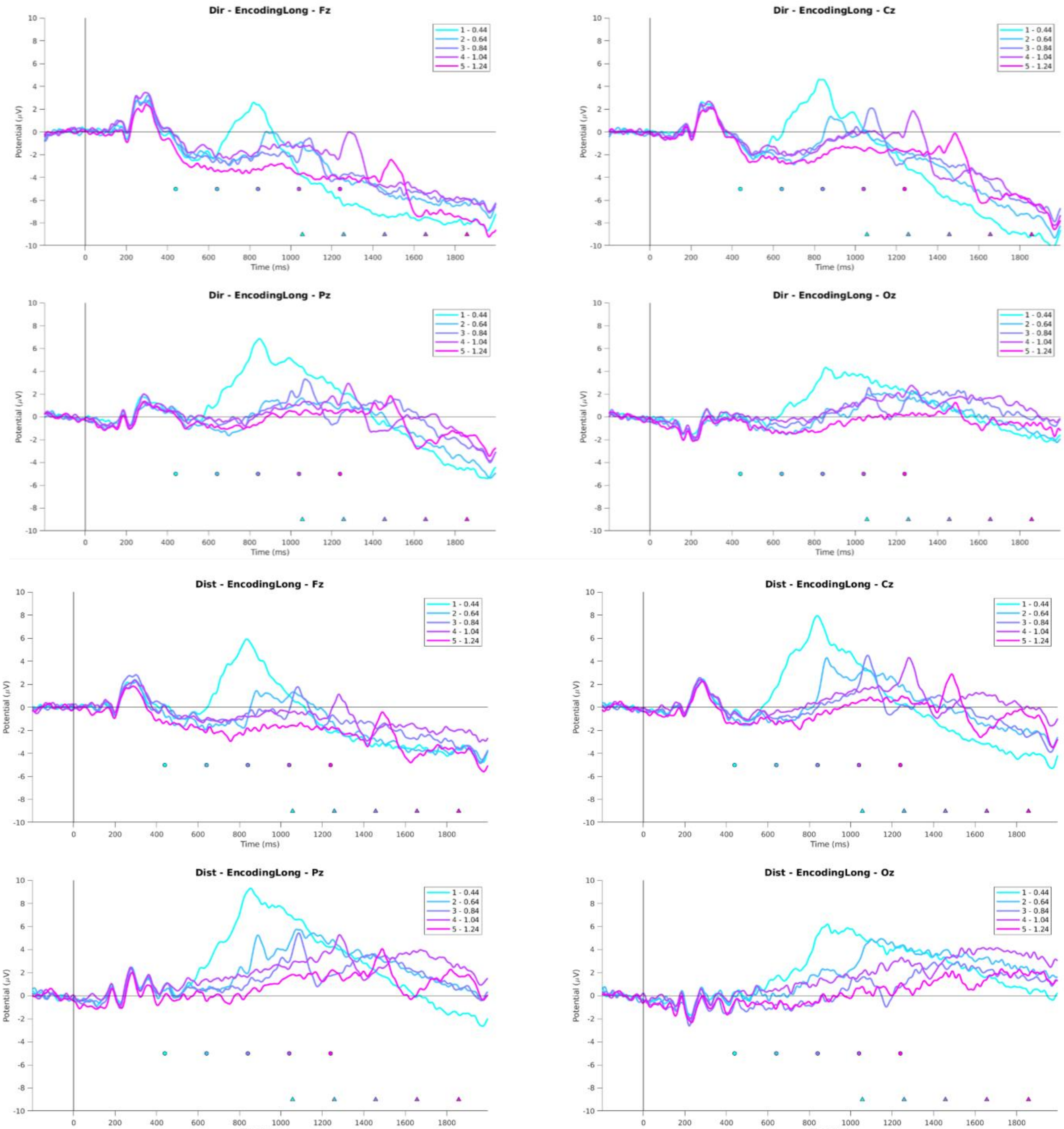

Figure S9: ERP epochs encompassing time encoding and decoding. Direction task in top two rows, Distance task in bottom two rows. Coloured dots represent probe offsets. To note, in the Decoding epochs, these time points represent the 0-ms point and the interval for baseline correction ranges from 50 ms before to 50 ms after probe offset. Coloured triangles represent the shortest presentation of the GO signal, relative to the corresponding probe offset. Normalizing ERPs around probe offset allows us to focus specifically on how experimental conditions affect neural activity during the decoding phase. This choice is motivated by the structure of the time-discrimination task, in which the interval between fixation onset, where no

condition-related differences are expected, and probe offset is long and variable, as it includes the cue and the probe and depends on probe duration (0.4–1.24 s). As a result, voltage values at probe offset can differ substantially across probe durations and conditions, as observed in the supplementary analyses (Fig. S9). These differences are difficult to interpret, as they may reflect slow fluctuations in electrical activity rather than genuine effects of condition or probe duration. For instance, for the shortest probe duration, probe offset coincides with later visual ERP components evoked by stimulus onset. By normalizing the ERP signal in a narrow window around probe offset (–50 to 50 ms), we remove these baseline differences and isolate neural processes occurring after probe offset, that is, during temporal decoding. Consistent with this rationale, we did not observe any reliable ERP effects of probe duration or condition during the encoding phase, indicating that advance knowledge of the upcoming movement in the Cued condition does not modulate encoding-related activity. Moreover, this approach is supported by previous work showing that ERP activity in the post-offset period, particularly 300–500 ms after stimulus offset, reflects neural processes underlying subjective duration judgments in temporal bisection tasks (Ofir & Landau, 2022).

Cued Vs Non-cued

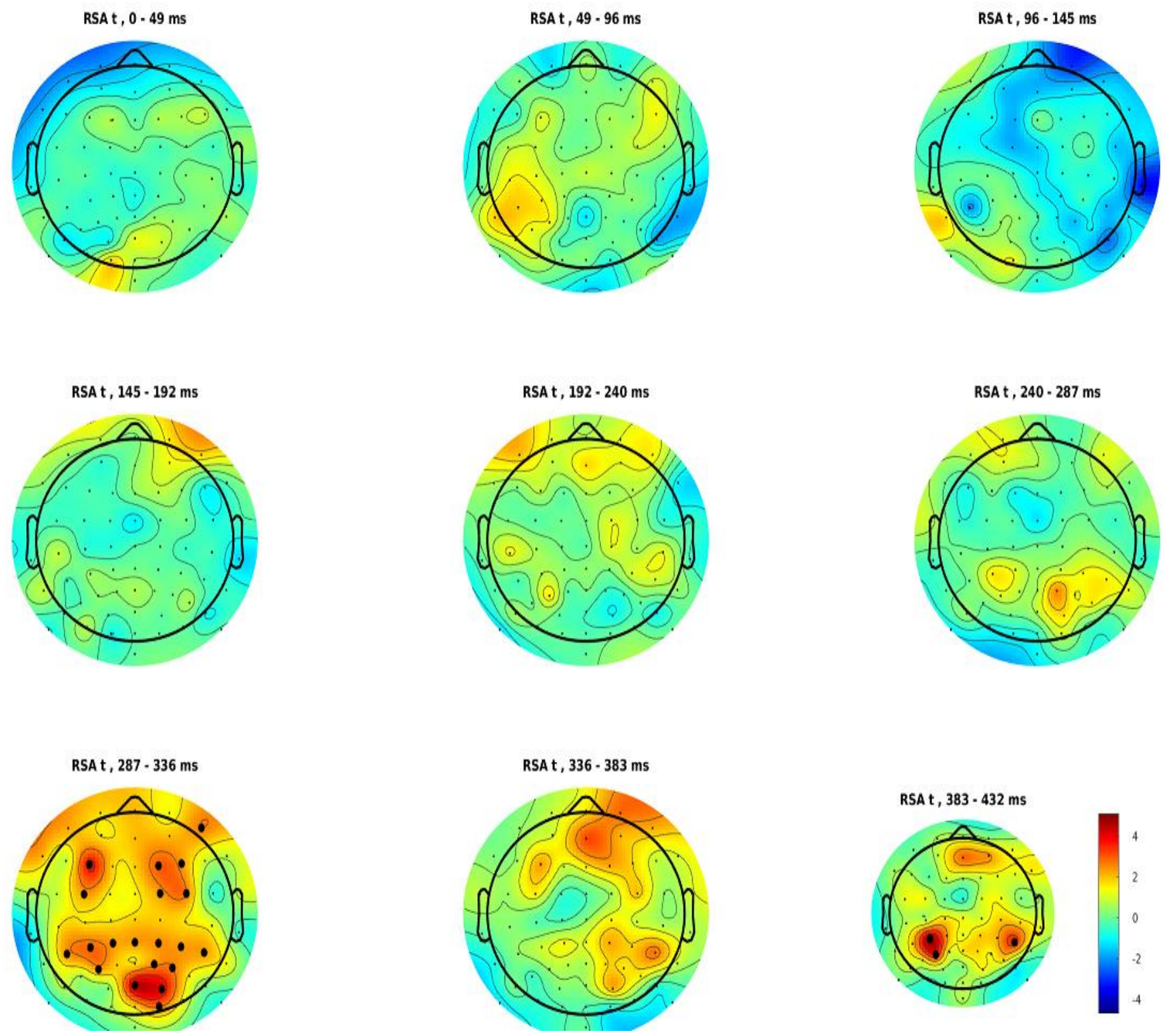

Cued Vs Sensory

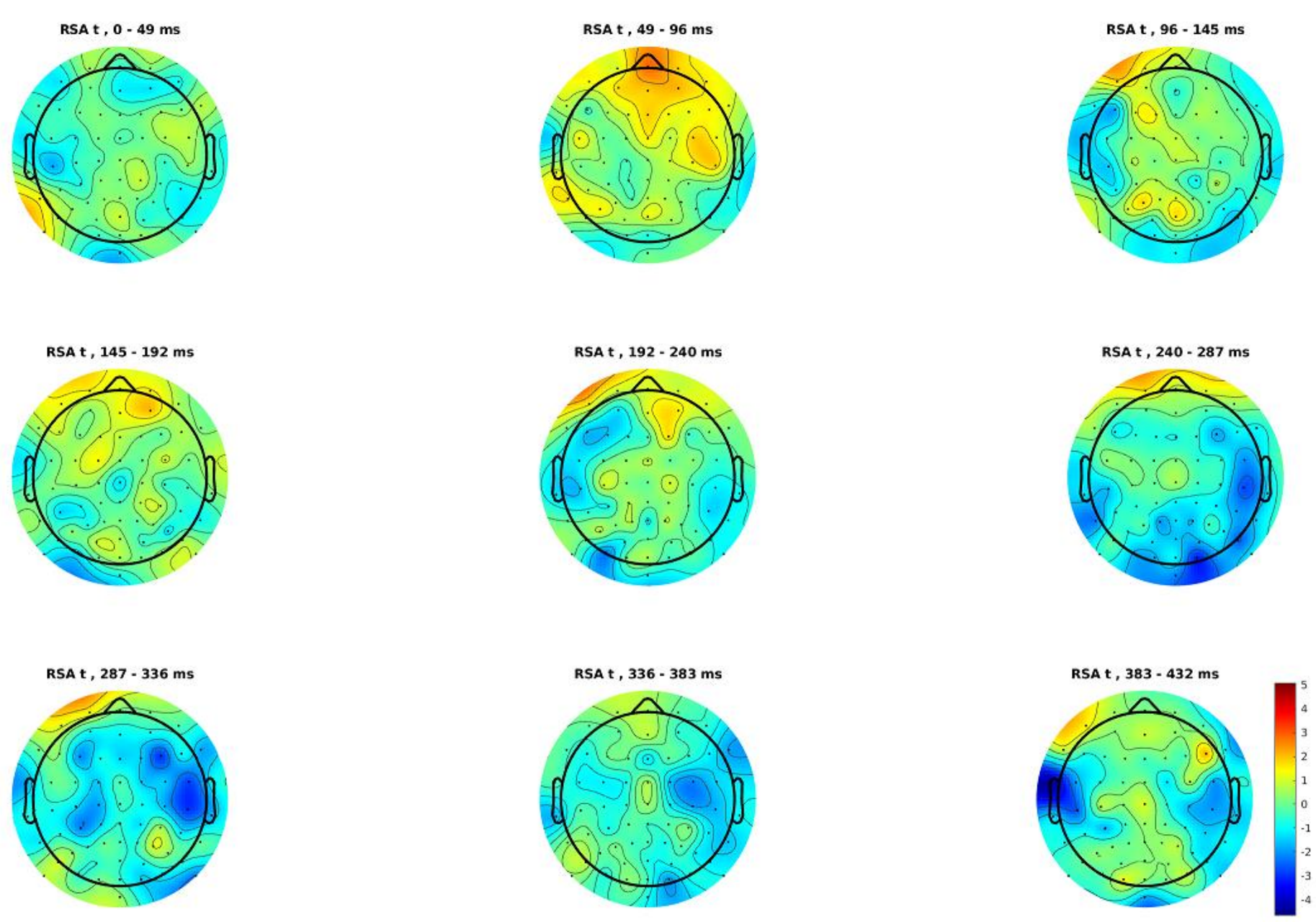

Figure S10: Direction Task - Encoding epoch RSA analysis (.43 sec following test stimulus onset), comparing Cued relative to Non-cued and Sensory conditions.

Cued Vs Non-cued

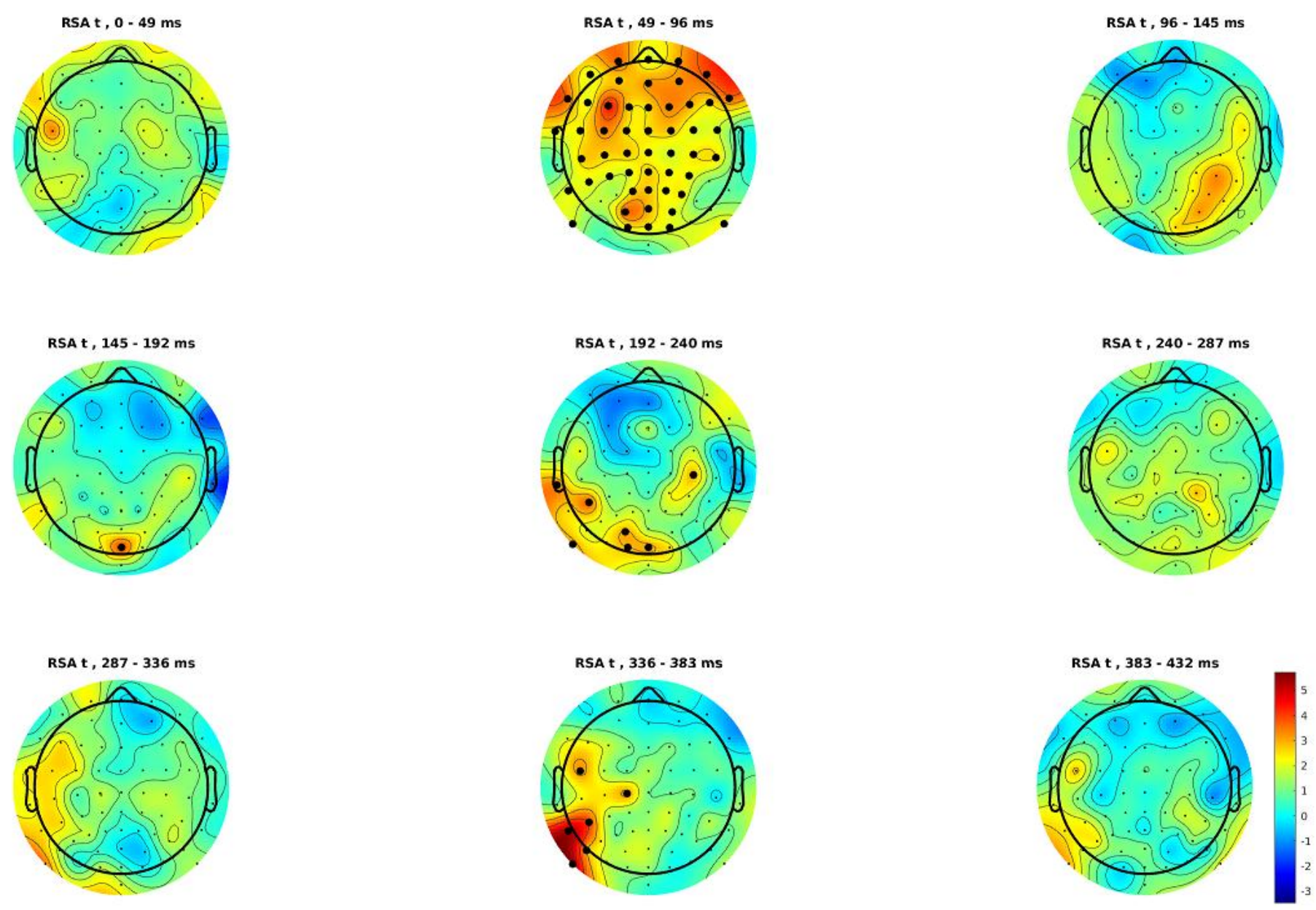

Cued Vs Sensory

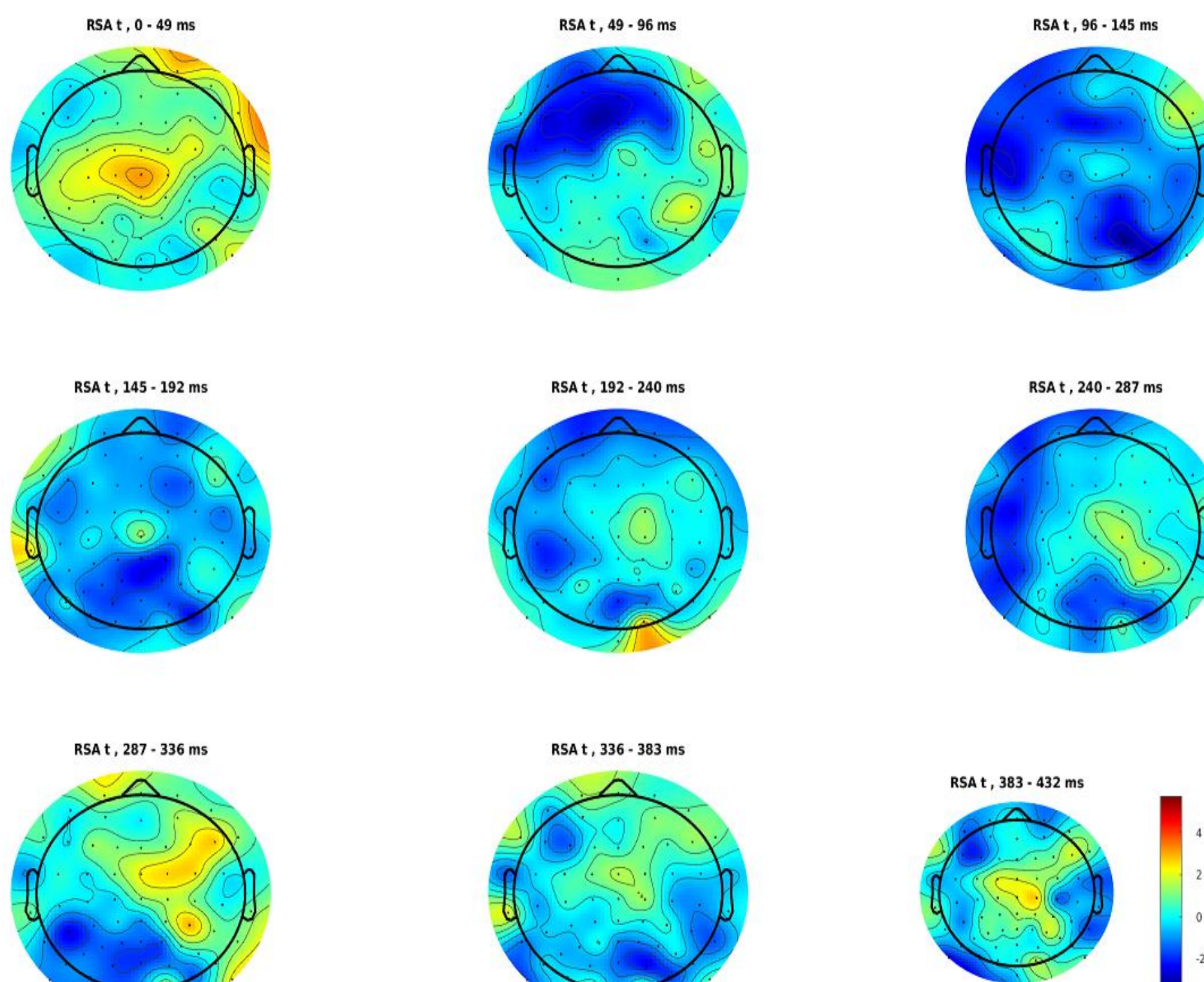

Figure S11: Distance Task - Encoding epoch RSA analysis (.43 sec following test stimulus onset), comparing Cued relative to Non-cued and Sensory conditions.

Cued Vs Non-cued

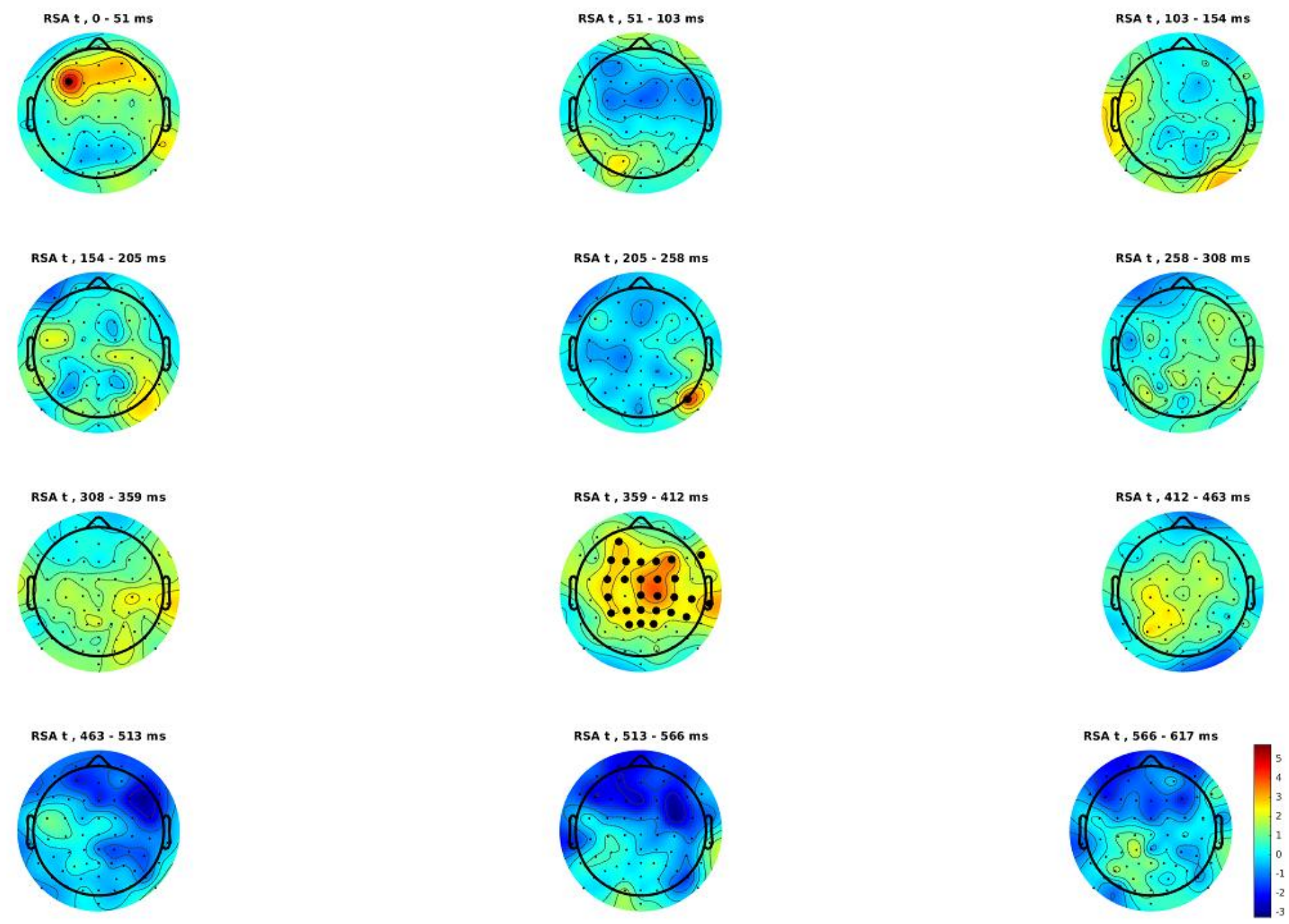

Cued Vs Sensory

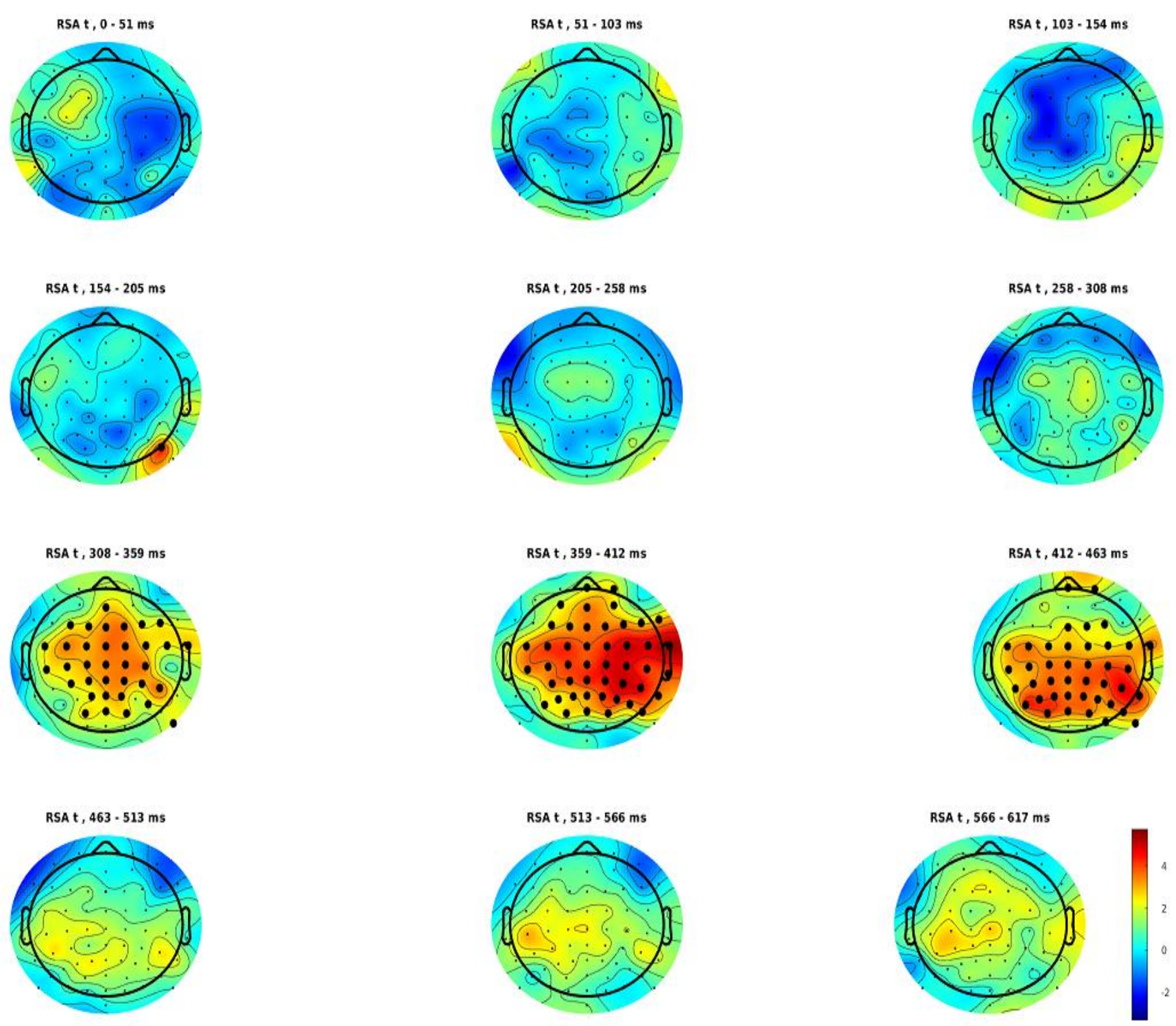

Figure S12: Direction Task - Decoding epoch RSA analysis (.62 sec following test stimulus onset), comparing Cued relative to Non-cued and Sensory conditions.

Cued Vs Non-cued

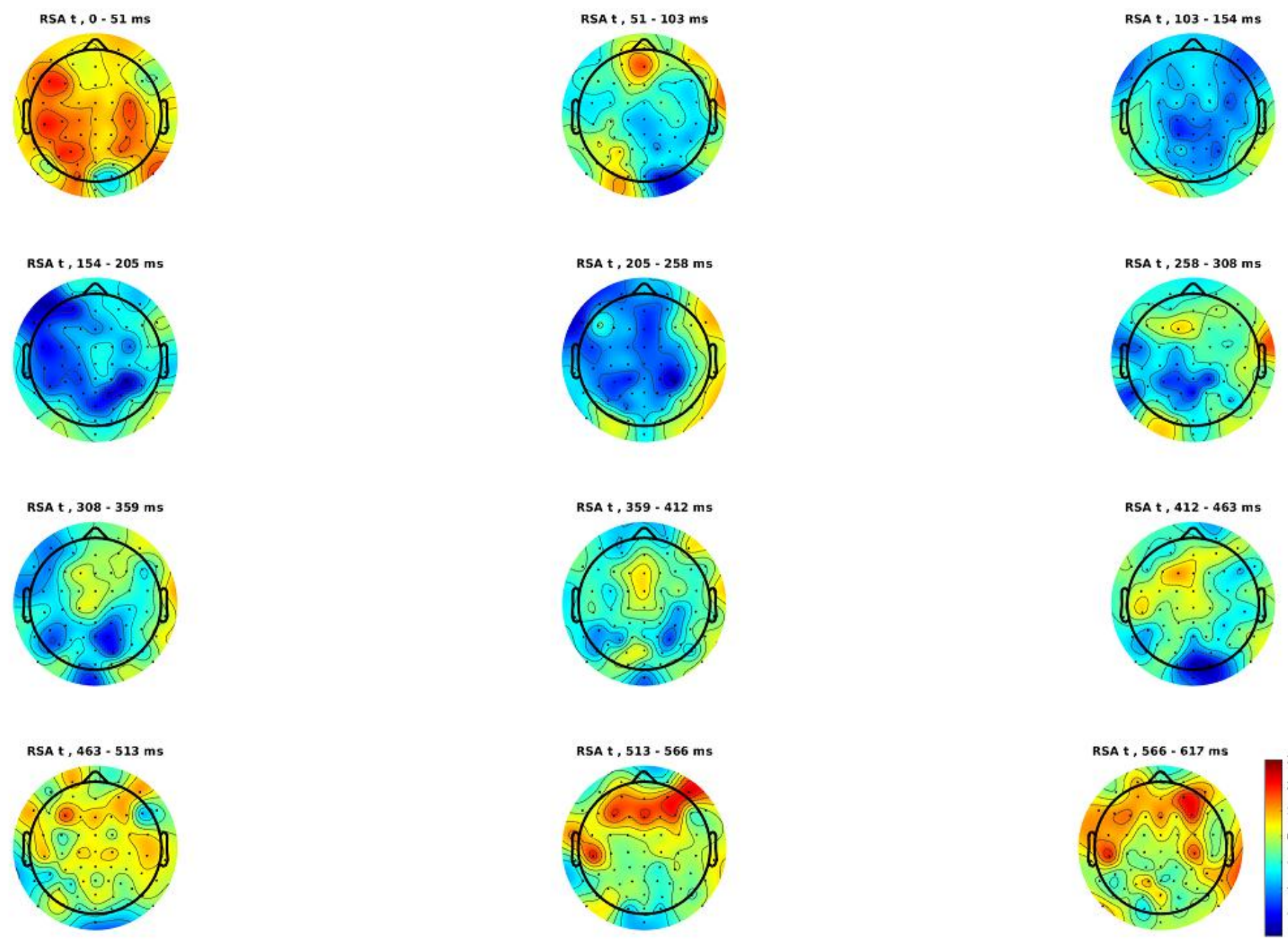

Cued Vs Sensory

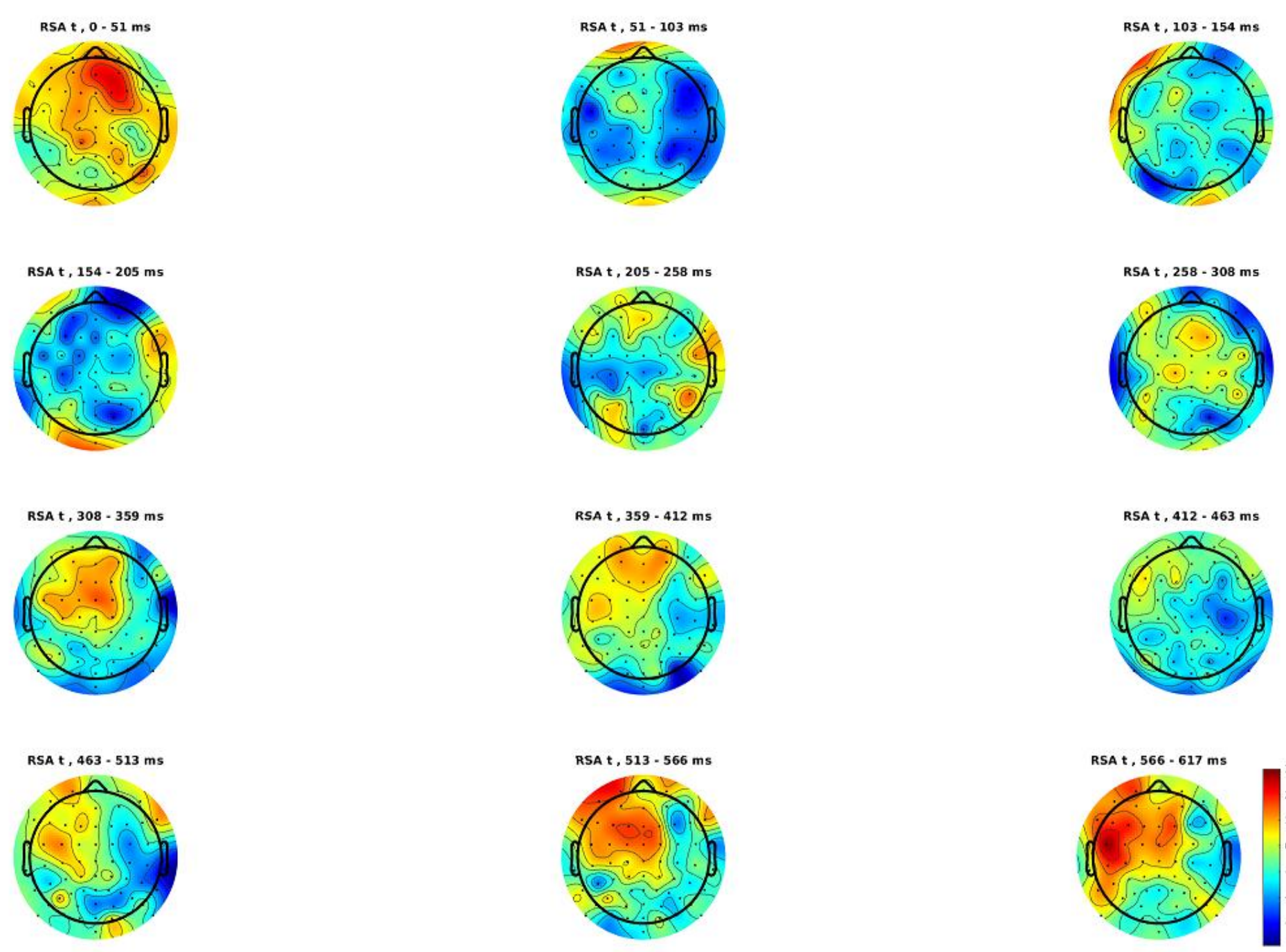

Figure S13: Distance Task - Decoding epoch RSA analysis (.62 sec following test stimulus onset), comparing Cued relative to Non-cued and Sensory conditions.

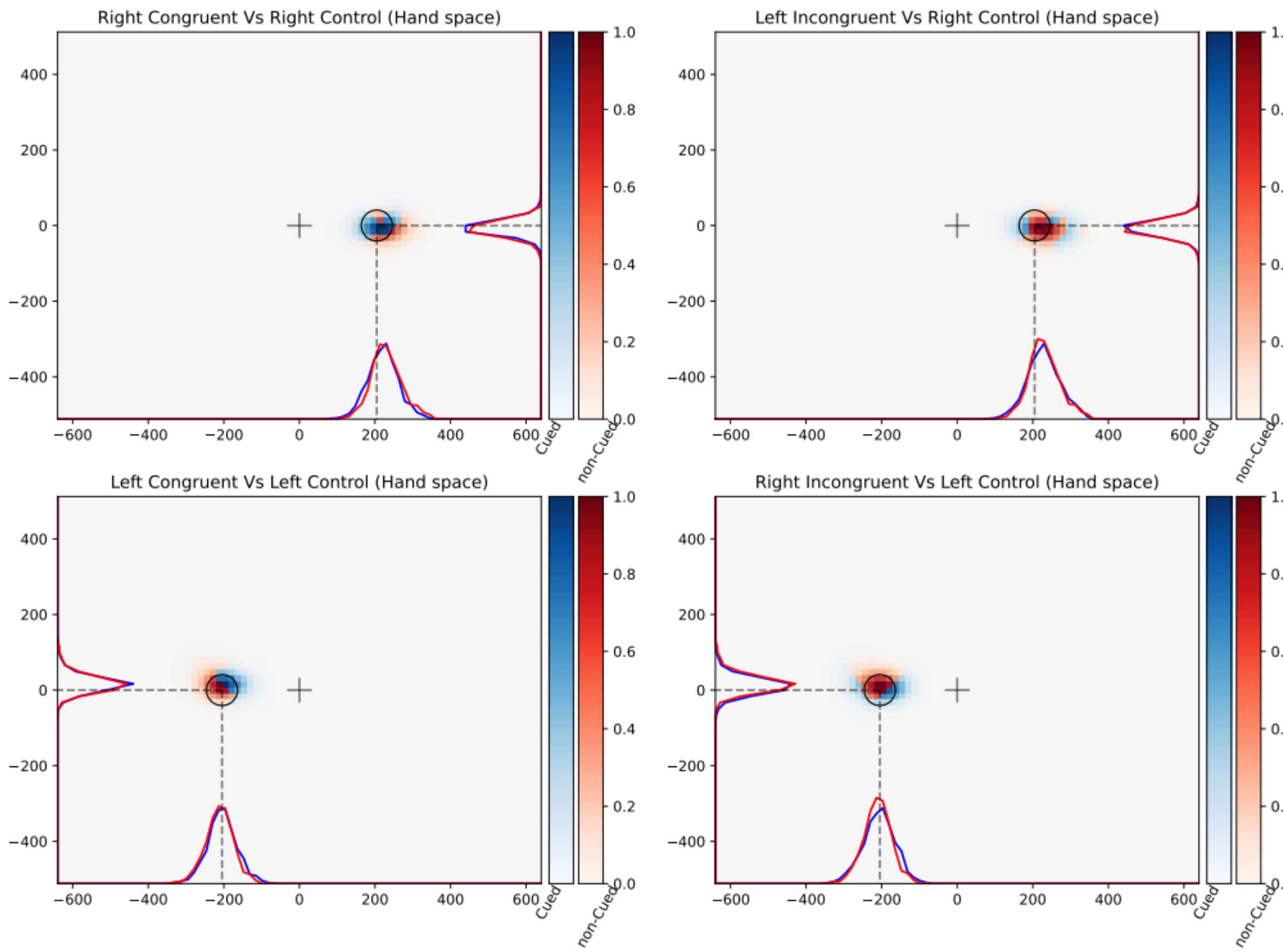

Figure S14: Direction Task - heatmaps depicting index finger reach end points in hand space (as recorded by the IR handtracker) across all participants and trials, for leftward or rightward actions. We manipulated camera to screen-space direction mappings in Cued trials (blue), yielding congruent (left column) and incongruent (right column) trials, presented in separate blocks of 20 trials. In congruent trials, a rightward (or leftward) target would require rightward (or leftward) hand movement, whereas in incongruent trials this mapping was reversed, with a rightward target requiring a leftward hand motion, and vice-versa. Non-cued trials (red) always had congruent hand to screen mappings, labelled as Control in plot titles. The central cross indicates the index starting position (aligned with the fixation cross on screen). The black circle represents the target position in hand space.

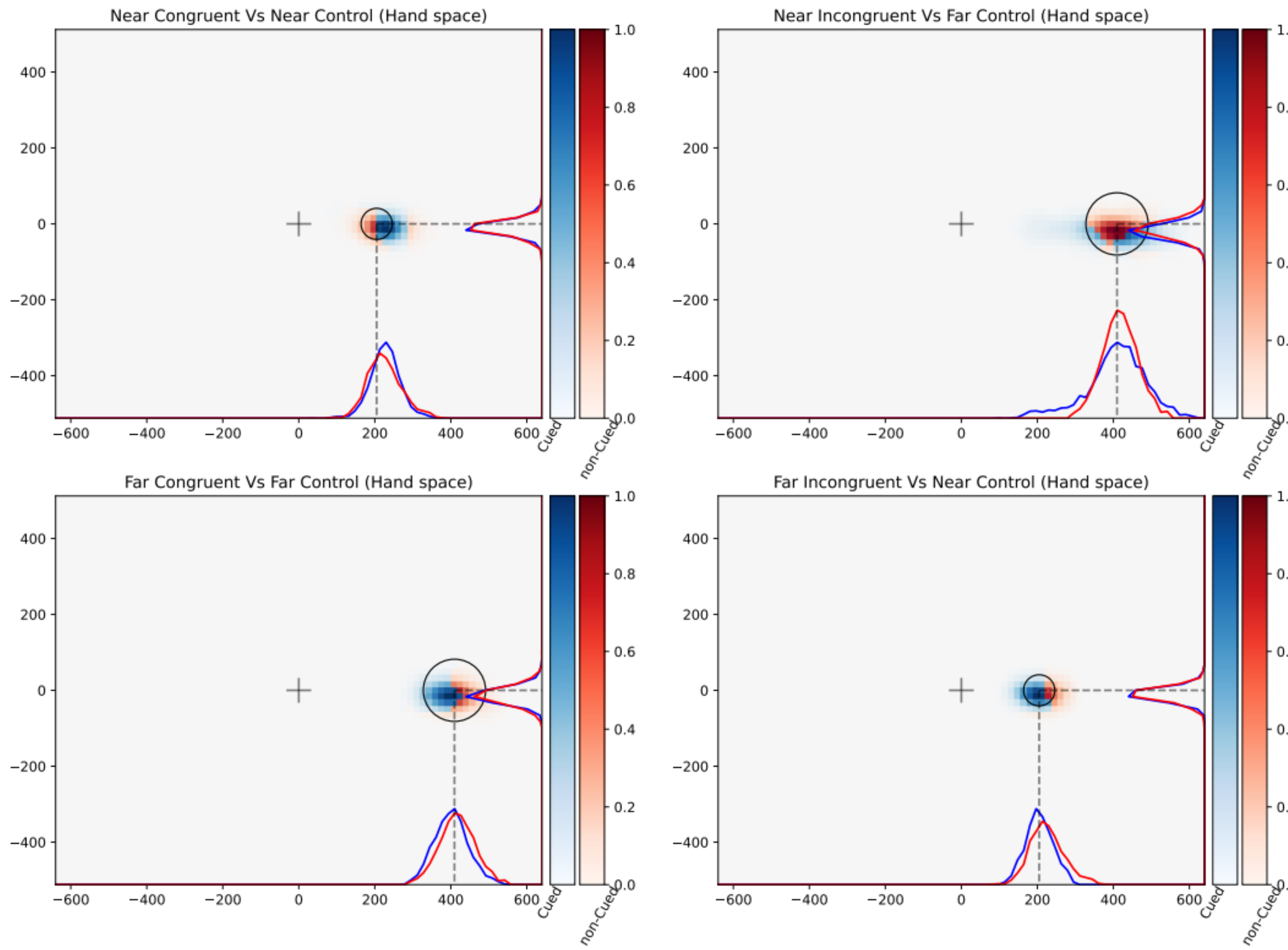

Figure S15: Distance Task - heatmaps depicting index finger reach end points in hand space across all participants and trials, for near or far targets. We manipulated camera to screen-space gain mappings in Cued trials (blue), yielding congruent (left column) and incongruent (right column) trials, presented in separate blocks of 20 trials. In congruent trials, a far (or near) target would require a large (or small) hand movement, whereas in incongruent trials this mapping was reversed, with a far target requiring a small hand movement, and vice-versa. Non-cued trials (red) always had congruent gain mappings, labelled as Control in plot titles. The central cross indicates the index starting position (aligned with the fixation cross on screen). The black circle represents the target position in hand space. Circle sizes reflect on-screen target size: target size was scaled based on distance in order to match task difficulty.

References

N. Ofir, A. N. Landau, Neural signatures of evidence accumulation in temporal decisions. Current Biology 32, 4093–4100 (2022).
